# Aquaculture ecosystem microbiome at the water-fish interface: the case-study of rainbow trout fed with *Tenebrio molitor* novel diets

**DOI:** 10.1186/s12866-023-02990-y

**Published:** 2023-09-06

**Authors:** Antonia Bruno, Anna Sandionigi, Antonella Panio, Simona Rimoldi, Flavio Orizio, Giulia Agostinetto, Imam Hasan, Laura Gasco, Genciana Terova, Massimo Labra

**Affiliations:** 1 ZooPlantLab, Biotechnology and Biosciences Department, University of Milano-Bicocca, Milan, Italy; 2 Quantia Consulting Srl, Milan, Italy; 3 Institute of Molecular Bioimaging and Physiology, National Research Council (IBFM-CNR), Milan, Italy; 4 Department of Biotechnology and Life Sciences, University of Insubria, Varese, Italy; 5 Department of Agricultural, Forest and Food Sciences, University of Turin, Torino, Italy

**Keywords:** Microbiome, Built environment, Aquaculture, Insect feed, Rainbow trout, 16S rRNA gene

## Abstract

**Background:**

Sustainable aquaculture relies on multiple factors, including water quality, fish diets, and farmed fish. Replacing fishmeal (FM) with alternative protein sources is key for improving sustainability in aquaculture and promoting fish health. Indeed, great research efforts have been made to evaluate novel feed formulations, focusing especially on the effects on the fish gut microbiome. Few studies have explored host-environment interactions. In the present study, we evaluated the influence of novel insect-based (*Tenebrio molitor*) fish diets on the microbiome at the water-fish interface in an engineered rainbow trout (*Oncorhynchus mykiss*) farming ecosystem. Using 16S rRNA gene metabarcoding, we comprehensively analyzed the microbiomes of water, tank biofilm, fish intestinal mucus, fish cutis, and feed samples.

**Results:**

Core microbiome analysis revealed the presence of a highly reduced core shared by all sample sources, constituted by *Aeromonas* spp., in both the control and novel feed test groups. Network analysis showed that samples were clustered based on the sample source, with no significant differences related to the feed formulation tested. Thus, the different diets did not seem to affect the environment (water and tank biofilm) and fish (cutis and intestinal mucus) microbiomes. To disentangle the contribution of feed at a finer scale, we performed a differential abundance analysis and observed differential enrichment/impoverishment in specific taxa, comparing the samples belonging to the control diet group and the insect-based diet group.

**Conclusions:**

Omic exploration of the water-fish interface exposes patterns that are otherwise undetected. These data demonstrate a link between the environment and fish and show that subtle but significant differences are caused by feed composition. Thus, the research presented here is a step towards positively influencing the aquaculture environment and its microbiome.

**Supplementary Information:**

The online version contains supplementary material available at 10.1186/s12866-023-02990-y.

## Background

Aquaculture is a millennial-old activity that has evolved slowly, often by building on traditional knowledge. Scientific progress in the twenty-first century brought unprecedented growth: production of aquatic animals in 2020 was more than 60% higher than the average in the 1990s, considerably outpacing world population growth, largely due to increasing aquaculture production. In 2020, fisheries and aquaculture production reached an all-time record of 214 million tonnes [1]. This stunning expansion presents several challenges that must be addressed to achieve the final aim of sustainable farming [2]. Sustainable aquaculture relies on multiple factors, including farmed fish, water quality, and feed formulations.

Considering the last aspect, replacing fishmeal with alternative protein sources is a key objective to improve sustainability in aquaculture and promote fish health. Insects appear to be a valid alternative to fishmeal because they are rich in nutrients, have a lower environmental impact than other plant-based protein sources, and constitute part of the natural diet of fish [3–5]. One of the most commonly used species of insects in aquafeeds is yellow mealworm (*Tenebrio molitor*). Different feeding trials have shown good growth in trout (*Oncorhynchus mykiss*) fed diets with different levels of FM substitution with *T. molitor* meal [6, 7].

Considerable research efforts have been made to evaluate novel feed formulations, focusing especially on the effects on the fish gut microbiome. Indeed, the boost of high-throughput DNA sequencing techniques (HTS) has allowed an in-depth biodiversity analysis of microbial communities, generating unprecedented knowledge about the composition of host-associated microbiomes, especially gut microbiomes. However, for a deep and extensive comprehension of aquaculture ecosystem dynamics, this is not sufficient because many interactions link host-associated and environmental microbial communities. Remarkably, there is still scarce information about the fish skin microbiome, which represents the primary barrier in constant contact with the aqueous environment, allowing the easy exchange of microbes with the surrounding water. It is affected by both environmental and fish species-dependent factors [8–11]. Furthermore, fish skin biodiversity is negatively affected by captivity, showing extensive shifts in microbial composition, with the environment and diet identified as the major drivers of change [12]. A second neglected contribution to aquaculture ecosystem health is that of the environment, despite the well-documented role of aquatic biodiversity in ecosystem equilibrium [13]. A recent study [11] evaluated how water and tank biofilm microbiomes influence the fish microbiome across three mucosal environments (the gill, skin, and digesta). The results of this study highlight how the aquaculture environment is a unique source of microbes that colonize fish and how this can influence fish health. Indeed, the aqua culture facility itself represents a built environment [14] that hosts its own microbiome, but further studies are needed to use this knowledge to design the built environment and modulate the microbial communities that are hosted for sustainable aquaculture.

Thus, recent scientific evidence suggests an interplay between different factors in aquaculture ecosystem health. In this context, the effects of novel feed formulations on the microbiome at the water-fish interface are poorly characterized. The purpose of this study was to evaluate the influence of insect-based novel feed formulations on the environmental and fish microbiomes, and therefore on fish health. This study comprehensively considered feed, water, tank biofilm, fish cutis, and intestinal mucus microbiome to disentangle the microbial structure at the water-fish interface in an engineered ecosystem, such as an aquaculture plant. An enhanced understanding of microbiome diversity and function in high-value farmed fish species, such as rainbow trout is required to sustain fish and environmental health.

## Methods

### Experimental design

Water, water tank biofilm, feed, skin mucus (hereafter named “cutis”) and intestinal mucus (hereafter named “mucus”) samples were collected from the experimental fishery facility of the Department of Agricultural, Forest, and Food Sciences of the University of Turin (Italy), located in Carmagnola (Turin, Italy).

Rainbow trout of 78.3 ± 6.24 g mean initial weight were randomly distributed into 400 L tanks (3 tanks/diet, 21 fish/tank). Tanks were supplied with untreated artesian well water at the constant temperature of 13 ± 1 °C, in a flow-through open system (tank water inflow: 8 L/min). The dissolved oxygen levels were measured every 2 weeks and ranged between 7.6 and 8.7 mg/L, whereas the pH was 7.5–7.6. A feeding trial was conducted for 22 weeks (April - September 2018) with isonitrogenous, isolipidic, and isoenergetic extruded experimental diets. Feed formulations consisted of increasing percentages of inclusion of partially defatted meal derived from *T. molitor* larvae (diet B: 5% inclusion, 25% fish meal replacement, diet C: 10% inclusion, 50% fish meal replacement, and diet D: 20% inclusion, 100% fish meal replacement). In control tanks, trout were fed with a diet without insect meal (diet A). Main ingredients and proximate composition of the diets are described in [6] and detailed in Additional_file_1 - Supplementary Table S01. During the first 8 weeks, fish were fed at 1.6% of the tank biomass and then, according to the fish growth and water temperature, the daily quantity of distributed feed was decreased to 1.4%. Fish were fed twice a day (at 8 am and at 3 pm), 6 days per week. Feed intake was monitored at each administration. In order to update the daily feeding rate, fish in the tanks were weighed in bulk every 14 days. Mortality was checked every day. The feeds were stored in a refrigerated room (6 °C) for the entire duration of the feeding trial. Feeding and farming condition details are reported in [6].

Water samples (1 L each) were collected in April, June, and September 2018, corresponding to the initial, intermediate, and final phases of the trial, from each tank and from the inlet (before entering the tanks) (three replicates). Before environmental DNA extraction, water sample replicas of each feed formulation were pooled and then 1 L of water was filtered by vertical (orthogonal) filtration using membrane filters with a pore size = 0.2 μm (47 mm diameter, nitrocellulose membrane filter, Millipore^TM^). Regarding the third sampling date, different porosity filters were used in series to cope with the increased water turbidity and to retain inorganic material (3 μm porosity, 47 mm diameter, nitrocellulose membrane filter, Millipore™), and to trap most bacteria (0.2 μm porosity, 47 mm diameter, nitrocellulose membrane filter, Millipore™). Moreover, we introduced a third filtration step for those environmental bacteria that are smaller in size than 0.2 μm and that filters normally used do not retain (0.1 μm porosity, 47 mm diameter, polycarbonate membrane filter, Millipore™).

Water tank biofilm samples were collected at the end of the trial (September 2018) from the internal surface of each tank using sterile swabs, scraping the tank surfaces on three different sides (dry swab, Gemini swabs and Labware).

Water filters and biofilm swabs were then stored at − 20 °C until DNA extraction to preserve microbiome integrity [15].

Skin and gut microbiota were collected as described in a previous publication of our group [16]. However, briefly, at the end of the trial, six fish/diet were sampled from the tanks in which trout were fed with diet A (devoid of insect meal) and diet D (with 20% of insect meal to replace 100% of fish meal). To obtain skin microbiota, the fish body was gently scraped using individually wrapped sterile cotton swabs with plastic shafts, whereas gut autochthonous microbiota was obtained by scraping the mucosa of the entire intestine except for the pyloric caeca. Samples were then processed using 200 µL of Xpedition Lysis/Stabilization Solution, as described in [17] and stored at room temperature for up to 24 h until bacterial DNA extraction.

### DNA extraction

All the instruments, if not disposable, were sterilized with sodium hypochlorite or autoclaved before use. Pre- and post-amplification phases were carried out in separate rooms, and every step was conducted in a laminar flow cabinet to avoid any possible contamination with exogenous DNA. We included negative controls to verify the absence of contamination during DNA extraction steps.

Environmental DNA (water and tank biofilm) was extracted from the filters obtained by water filtration using a DNeasy® PowerWater® Kit (Qiagen, Italy), following the manufacturer’s protocol.

DNA from feed, cutis, and intestinal mucus samples was previously extracted by Terova et al. using the DNeasyPowerSoil® Kit (Qiagen, Italy) [16].

Total DNA was checked for concentration and purity using a Qubit 2.0 Fluorometer and a Qubit dsDNA HS Assay Kit (Invitrogen, Carlsbad, California, United States).

### Library preparation and high-throughput DNA sequencing

The V3–V4 hypervariable regions of the 16S ribosomal RNA (rRNA) gene were amplified with S-D-Bact-0341-b-S-17, 5′-CCTACGGGNGGCWGCAG-3′ and S-D-Bact-0785-a-A-21, 5′-GACTACHVGGGTATCTAATCC-3′ primer pairs with overhanging adapters, according to the 16S Metagenomic Sequencing Library Preparation protocol, Part # 15,044,223 Rev. B (Illumina, SanDiego, CA, United States). Negative controls were used to verify the absence of exogenous DNA contamination. The length of the amplified DNA sequence was 550 bp. Amplicon PCR products were checked using capillary electrophoresis with a QIAxcel Advanced System (Qiagen) to verify the amplification and the correct length of the amplicon. Index-PCR and Illumina MiSeq v3 2 × 300 paired-end sequencing were carried out by “Center for Translational Genomics and Bioinformatics” (CTGB, San Raffaele Institute, Milan) in the case of water and tank biofilm samples and at BMR Genomics (Padova, Italy) for all the remaining samples.

### DNA sequences analyses

Raw paired-end FASTQ reads were imported into the Quantitative Insights Into Microbial Ecology 2 program (QIIME2, ver. 2020.6) [18, 19], and demultiplexed native plugins. Raw reads were subsequently deposited in the European Nucleotide Archive (ENA) (see Data Availability paragraph). The Divisive Amplicon Denoising Algorithm 2 (DADA2) [20] was used to filter, trim, denoise, and merge pairs of the obtained reads. The chimeric sequences were removed using the consensus method. The taxonomic assignment of the ASVs calculated was carried out using the feature-classifier plugin [21] implemented in QIIME2 against the SILVA SSU non-redundant database (138 release), adopting a consensus confidence threshold of 0.8. Reads of mitochondrial or eukaryotic origins were excluded.

Rarefaction curves were calculated, and taxa-bar plots were generated using the QIIME2 dedicated plugin taxa [22].

To estimate the effect of the different sampling sources and feed conditions on alpha diversity, the Observed ASVs, Shannon Index, and Inverse Simpson Index [23, 24] were calculated. The Kruskal-Wallis H test for all and pairwise tests were used to compare the groups. When multiple tests were applied, we used the Benjamini and Hochberg correction, and the obtained q-value was reported in the text [25, 26].

To visualize the distribution of shared ASVs among different sample sources, we plotted a Venn diagram using the VennDiagram R package.

We adopted an ordination approach to explore the structure of microbial communities; specifically, we used Non-metric Multidimensional Scaling (NMDS) implemented in the phyloseq R package (McMurdie and Holmes, 2013). Bray-Curtis dissimilarity [27] was used to perform community analyses (beta diversity), evenly sampled at 9000 reads per sample, using the core-metrics-phylogenetic QIIME2 plugin. Samples with less than this threshold were excluded from downstream analyses.

Statistical significance among groups (sample source and feed condition) was determined by the ADONIS (permutation-based ANOVA, PerMANOVA) test [28] with 1000 permutation-based Bray-Curtis. PerMANOVA Pairwise contrast was performed, and the Benjamini-Hochberg FDR correction was used to calculate q-values. The test was performed using the beta group-significance QIIME2 implemented plugin based on the adonis function in vegan R package [29].

A differential abundance analysis was carried out using negative binomial generalized linear models [30] to estimate differences between groups considering the relative abundance of ASVs assigned to the taxonomic rank of the genus.

### Water samples bacterial load and chemical analyses

Quantitative real-time PCR amplification assay (qPCR) was performed with AB 7500 (Applied Biosystems) to quantify bacterial DNA in water and tank biofilm samples and verify the absence of amplification reaction inhibitors targeting the same 16S rDNA region chosen for high-throughput DNA sequencing, as described in [31]. Briefly, qPCR conditions included an initial denaturation at 95 °C for 10 min, followed by 40 cycles of denaturation at 95 °C for 15 s and annealing and elongation at 55 °C for 1 min. The final dissociation stage was then performed. Amplification reaction consisted of 5.0 µl SsoFast EvaGreen Supermix with Low ROX (Bio-Rad S.r.l., Italy), 0.1 µl each 10 µmol/L primer solution, 2 µl DNA sample, and 2.8 µl of Milli-Q water. All samples and negative controls (no template) were run in triplicates. Threshold Cycle (Ct) values were converted to counts (DNA copies) [31]. Statistical analyses were performed using RStudio software version 1.0.44 (© RStudio, Inc.). Data of the DNA counts were log-transformed, and a linear mixed-effects model (LME) was used, using the lme4 package [32], with the DNA counts as a response variable, while different tanks and sampling dates were taken into consideration as fixed effects, and random effects were the PCR replicates available for each sample. The significance of the fixed variables was evaluated using the drop1 function.

Heterotrophic cultivable bacteria were quantified in each collected water. One milliliter of water was placed on a solid non-selective nutrient medium (Plate Count Agar, PCA). The growth occurred in duplicate at temperatures of 22 and 37 °C, and colony forming unit (CFU) enumeration was carried out by visual observation, according to D. Lgs. n. 31 of February 2, 2001 (implementing the European Directive 98/83/CEE) and after seven days of incubation, to facilitate environmental bacterial growth.

For each water sample, total nitrogen, nitrites, nitrates, ammoniacal nitrogen, and phosphate were measured using a spectrophotometer (Spectroquant Pharo 300; Merck). The pH and conductivity values were also recorded. Analyses were performed using the following kits, according to the manufacturer’s instructions: ammonium test photometric method NH4-N, nitrate test photometric method NO3-N, nitrite test photometric method NO2-N, total nitrogen test photometric method, phosphate test photometric method PO4-P, and Merck Spectroquant®. Statistical analyses were performed for values > 0 mg/L, using RStudio software (© RStudio, Inc.), applying a linear model (LM), where the effect of the different tanks was tested (fixed effect) on the concentration of the different chemical parameters (response variable), using the lme4 R package [32]. The significance of the results was assessed using the drop1 function.

## Results

### Water samples characteristics

The microbiological results of water samples are detailed in Additional_file_1 - Supplementary Data S01, Additional_file_1 - Figures S01 and S02, Additional_file_1 - Tables S02 and S03.

Chemical characteristics are reported in Additional_file_1 - Supplementary Data S02, Figure S03, and Table S04.

Overall, no significant differences in microbiological and chemical parameters were recorded between the different feeding formulations. However, temporal variation (considering sampling date) was reported when measuring nitrogen compounds and bacterial load by 16S rRNA gene qPCR assay. The bacterial load on the third sampling date was significantly higher than that on the first and second sampling dates. Nitrate and total nitrogen concentrations showed significant variations in September 2018 and April 2018, respectively.

### High-throughput DNA sequencing analysis

A total of 61 samples (15 water samples, 14 tank biofilm samples, 12 gut mucus samples, 12 cutis samples, and 8 feed samples) were analyzed to explore their microbiomes.

From the high-throughput DNA sequencing of the hypervariable regions V3-V4 of the 16S rRNA gene, after quality filtering, merging reads, and chimera removal of the two Illumina runs, we obtained 6,105,976 sequences, with a median frequency of 64,547 reads per sample. We obtained 12,798 ASVs (amplicon sequence variants, [33] and in order to clean the data from spourius ASVs, a subset to min 50X reads per ASV was created, obtaining 2948 ASVs.

Considering the reads distribution among samples, water samples had the highest number of reads (even without considering INST_A, which had an exceptional number of reads), followed by tank biofilm samples, and feed samples had the lowest (Additional_file_1 - Supplementary Fig. S04).

### Microbiome diversity composition and distribution

Microbiome biodiversity and composition were evaluated considering different sample sources and feed conditions.

Two samples (236032F221730: feed D; T-01 C: tank biofilm A) were removed from subsequent analyses because of their low number of reads (8919 and 141, respectively).

**Fig. 1:**
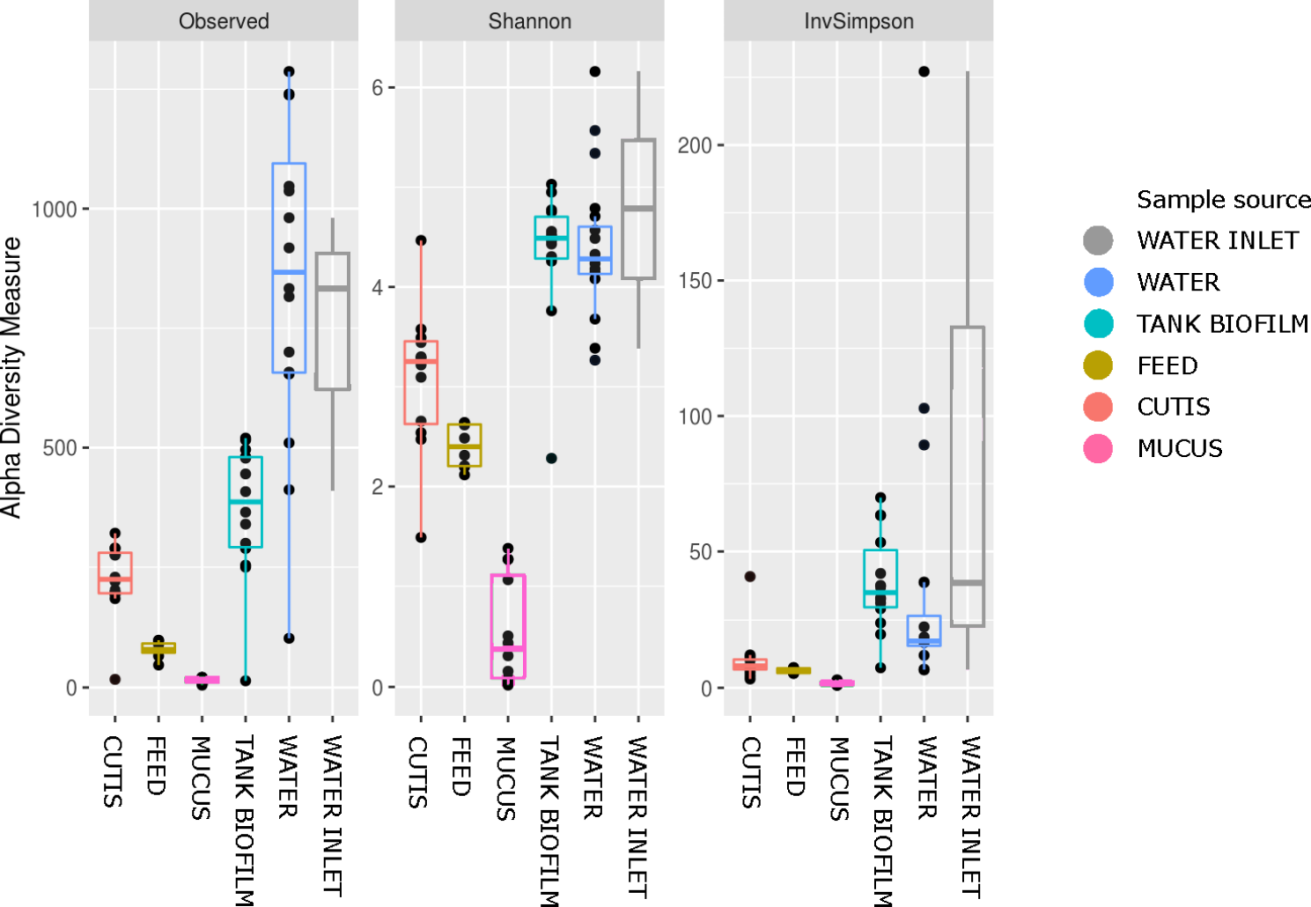
Alpha diversity boxplot for sample sources, considering the Observed ASVs, Shannon Diversity Index, and Inverse Simpson Index

Three main indices were calculated to describe the diversity within the samples (alpha diversity) for each sample source: the Observed ASVs, Shannon index, and Inverse Simpson index (Fig. 1).

Considering all the metrics, water samples, in particular inlet water, showed the highest alpha diversity, followed by tank biofilm samples. The lowest alpha diversity was measured in the mucus and feed samples (Fig. 1). Considering all different sample sources, the Kruskal-Wallis H test was significant (p < 0.05).

Taxonomy proportions assigned to the ASV were 97%, 96%, 89%, 77%, and 56% for the taxonomic ranks of Phylum, Class, Order, Family, and Genus, respectively. Taxonomic analysis revealed that most of the sequences in all samples were associated with the phyla *Proteobacteria* (53.5%), *Bacteroidota* (18.9%), and *Firmicutes* (17.5%). Looking inside the class rank, we found members mainly belonging to *Gammaproteobacteria*, *Bacteroidia*, and *Bacilli* (44.5%, 18.9%, and 14.7%, respectively). Concordantly, the three most abundant families reported were *Mycoplasmataceae* (*Firmicutes*), *Comamonadaceae* (*Proteobacteria*), and *Flavobacteriaceae* (*Bacteroidota*) (11.9%, 10.7%, and 8.2%, respectively) (Additional_file_2 - Supplementary Data S03).

The top five genera assigned were *Mycoplasma* (*Firmicutes*), 11.9% reads; *Flavobacterium* (*Bacteroidia*), 8.2% reads; *Pseudomonas* (*Proteobacteria*), 5.6% reads; *Acinetobacter* (*Proteobacteria*), 5.4% reads; and *Rheinheimera* (*Proteobacteria*), 5% reads. Notably, we reported the presence of Gracilibacteria (class rank, Patescibacteria phylum) and *Cyanobacteria* (phylum rank) in all sample sources except for feed and mucus, regardless of the feeding administered.

**Fig. 2:**
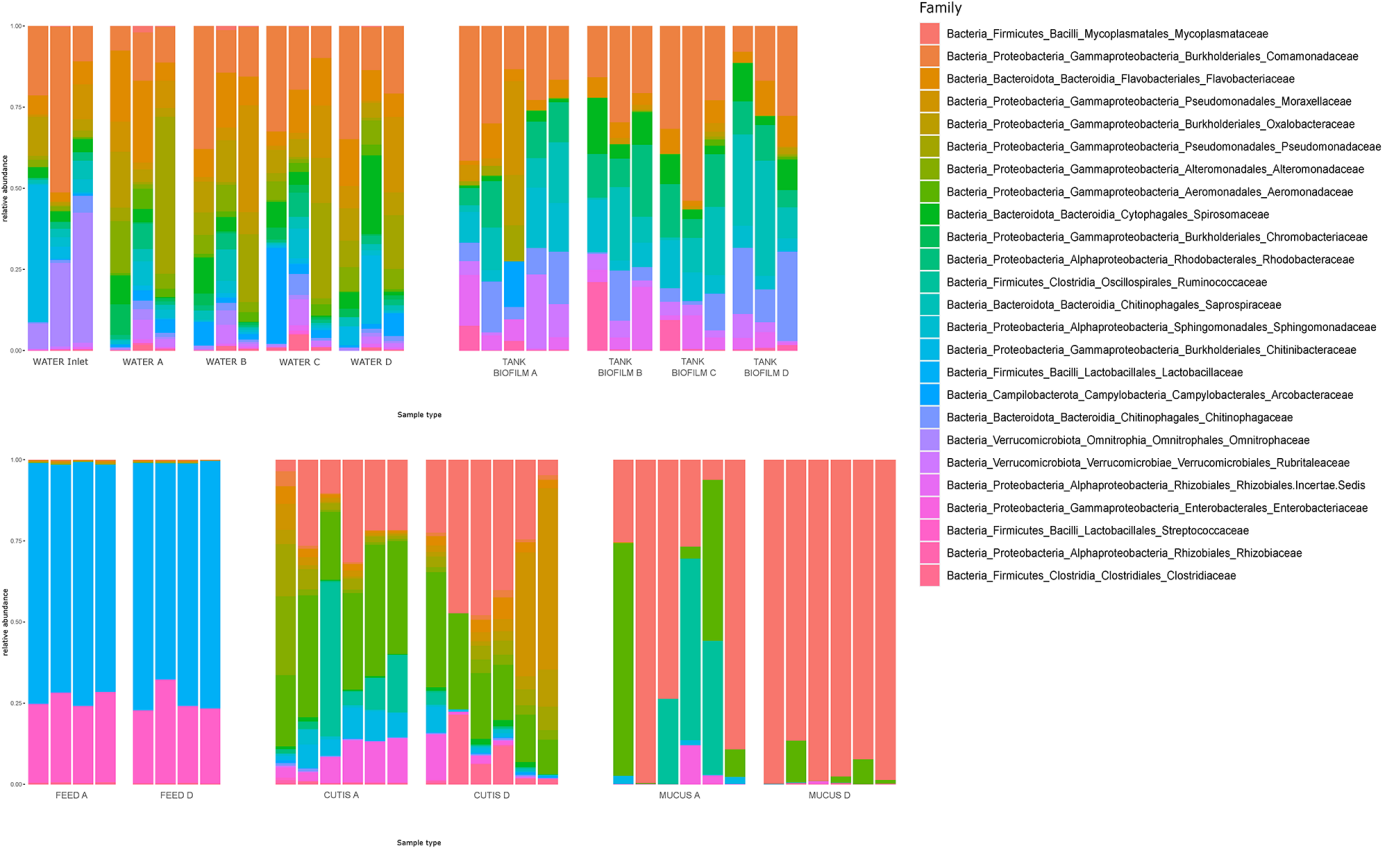
Bar chart depicting the relative abundance and distribution of the 30 most abundant bacterial families. Each bar represents a sample, and the y-axis indicates the relative abundance (from 0 to 100%)

Bar chart representation highlights the distribution of sequences across samples, assigned to the taxonomic rank of the Family (Fig. 2).

**Fig. 3:**
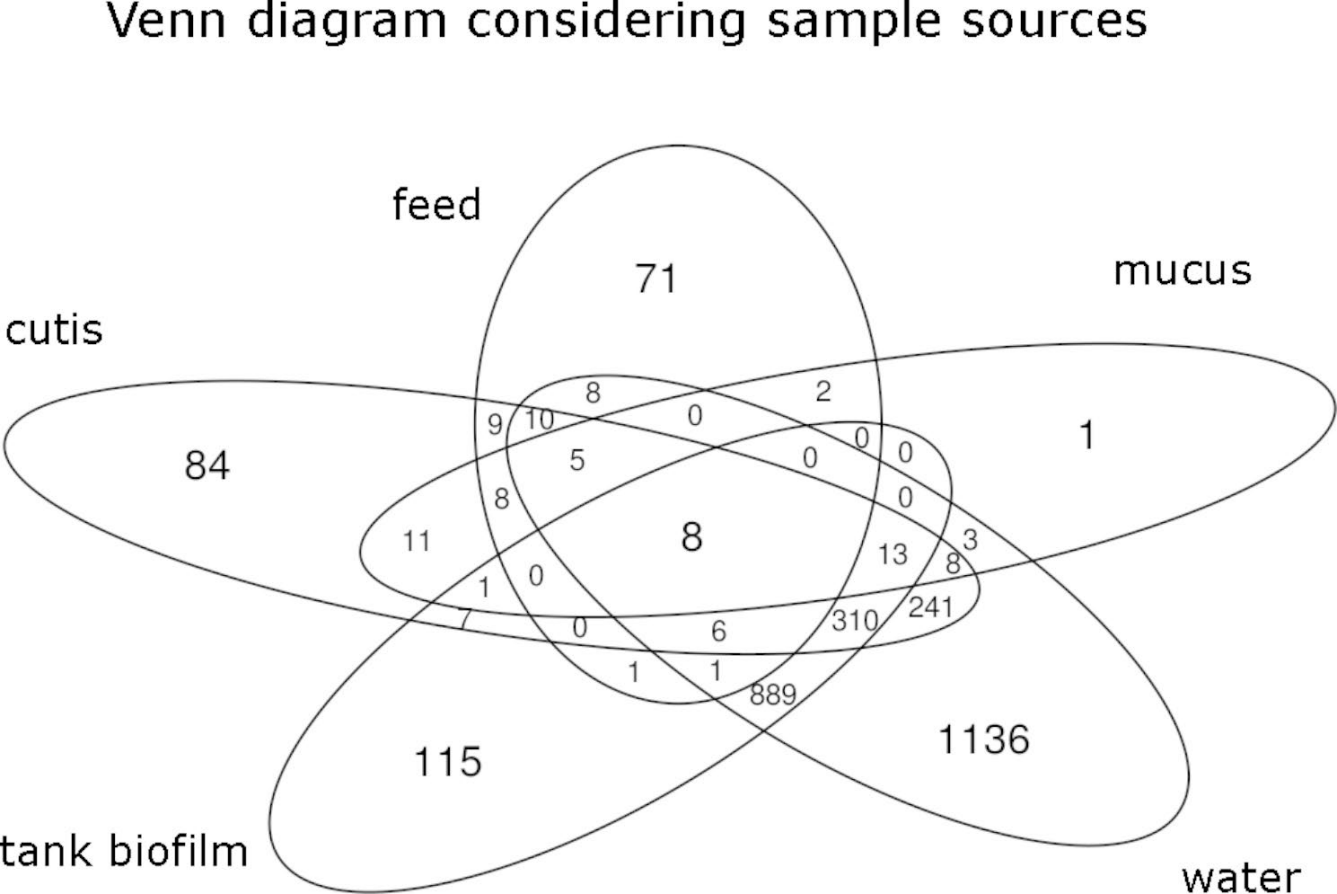
Venn diagram representing shared ASVs among sample sources

The distribution of shared ASVs among the different sample sources is shown in the Venn diagram (Fig. 3).

Eight ASVs were shared by all samples, assigned to the genera *Cutibacterium*, *Streptococcus*, *Mycoplasma*, *Methylobacterium-Methylorubrum* (uncultured bacterium), *Acinetobater*, *Aeromonas*, and *Enhydrobacter* (uncultured bacterium).

Notably, mucus samples had only one unique ASV, in contrast to the water samples, which had 1136 exclusive ASVs. The tank biofilm and water samples shared 1227 ASVs. The cutis and water samples had in common 601 ASVs. 37 ASVs were shared between the mucus and water samples.

Notably, a single ASV, assigned to the genus *Mycoplasma*, was retrieved (with > 800 reads) from 35 samples, all belonging to cutis and mucus samples.

### Core microbiome analyses

The core microbiome was calculated considering samples per feed formulation and assigned at the taxonomic rank of the genus. In the case of both feed formulation A (control) and feed formulation D samples, *Aeromonas* was the only genus shared by at least 90% of the samples, regardless of the sample source. In 80% of the samples characterized by feed formulation A, the shared genera were *Aeromonas*, *Mycoplasma*, *Acinetobacter*, and *Pseudomonas*, whereas in the case of formulation D, they were *Aeromona*s and *Mycoplasma*, regardless of the sample source.

### Microbial community analyses

The abundance of each ASV within and across samples is shown with a heatmap (Additional_file_1 - Supplementary Fig. S05). Interestingly, patterns of abundance were also observed. For instance, feed samples showed a high abundance of ASVs assigned to *Lactobacillus*, *Streptococcus*, *Photobacterium*, and *Weissella* (*Lactobacillales*), all Gram-positive bacteria with the exception of *Photobacterium*. *Mycoplasma* was found to dominate in the cutis and mucus samples. A cluster of ASVs was more abundant in water and tank biofilm samples and was assigned to *Sphaerotilus* (*Burkholderiales*), *Bacteroidetes* bacterium OLB8, *Hydrogenophaga (Burkholderiales*), *Haliscomenobacter* (*Chitinophagales*), *Rhodoferax* (*Burkholderiales*), *Phreatobacter* (*Rhizobiales*), and other uncultured bacteria.

**Fig. 4:**
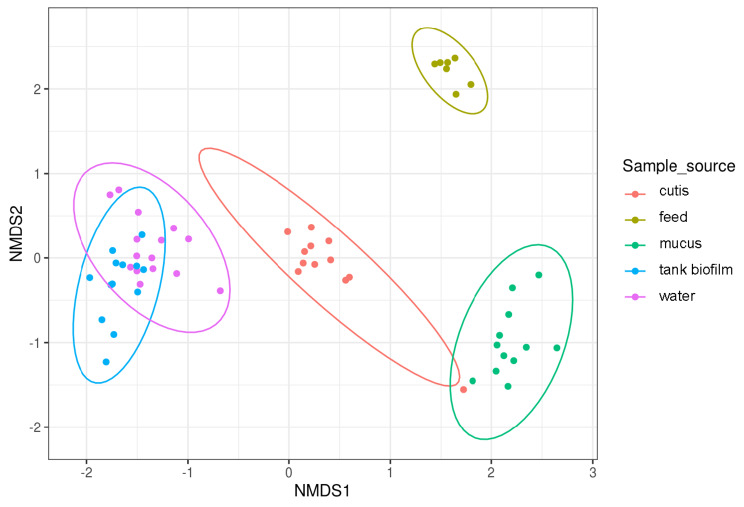
NMDS based on the Bray-Curtis metric considering the sample source

According to the NMDS plot drawn on Bray-Curtis dissimilarity, samples were clustered based on their source. Feed samples clustered together and were clearly separated from the other groups as well as cutis, mucus, water, and tank biofilm samples. No complete overlap was observed between the water and tank biofilm samples (Fig. 4).

Paired ADONIS (1000 permutations) results showed that significant differences existed among the different sample sources (Additional_file_1 - Supplementary Table S05).

Considering the different feed formulations for each type of sample (“sample type”), no significant difference was observed (but higher differences were observed between feeding formulations for feed and mucus samples) (Additional_file_1 - Supplementary Table S06).

Moreover, the NMDS plot of taxa distribution across samples showed that some phyla were differentially distributed according to the sample source (Additional_file_1 - Supplementary Fig. S06). Indeed, if *Firmicutes* and *Proteobacteria* were found in almost all samples, regardless of the sample source, Patescibacteria, *Myxococcota*, *Nitrospirota*, and *Verrucomicrobiota* were typical of water and tank biofilm samples. *Bacteroidota* were found in all sample sources, but not in the mucus samples.

### Network analyses

**Fig. 5:**
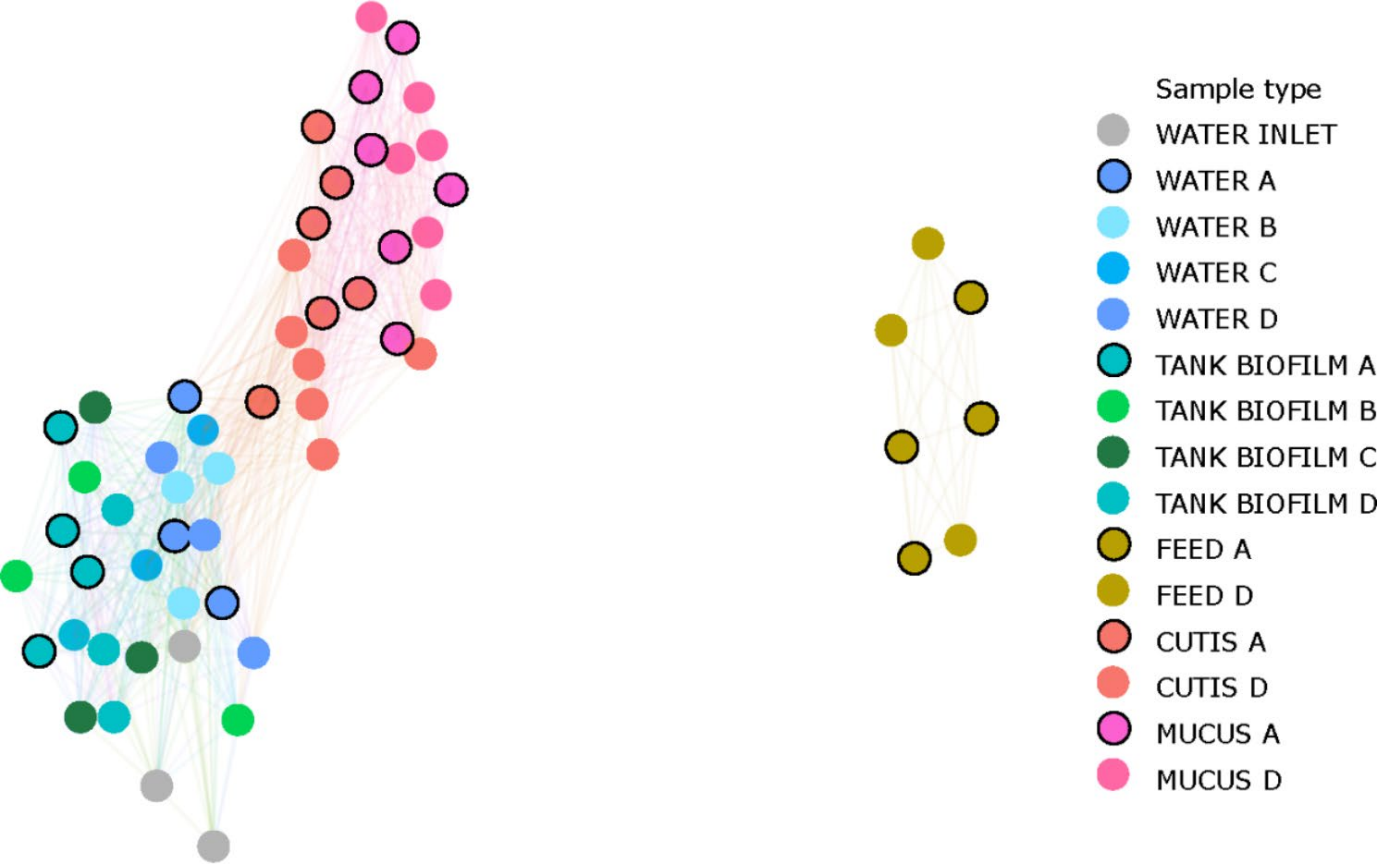
Network graph based on the Bray-Curtis dissimilarity. Sample types are shown

The construction of a network analysis (based on Bray-Curtis dissimilarity) allowed us to highlight whether any correlation existed among different sample sources and sample types (Fig. 5).

The network displayed a structure with a major subgraph comprising the majority of the samples and a minor subgraph made using only feed samples. Furthermore, a correlation between the samples belonging to cutis and mucus, on the one hand, and water and tank biofilms, was highlighted within the network. Different feeding formulations were not the drivers of the correlations.

### Differential abundance analysis

Thus, to deepen the analysis and disentangle any contribution of feeding formulations at a finer scale, a differential abundance analysis was carried out using negative binomial generalized linear models.

**Fig. 6:**
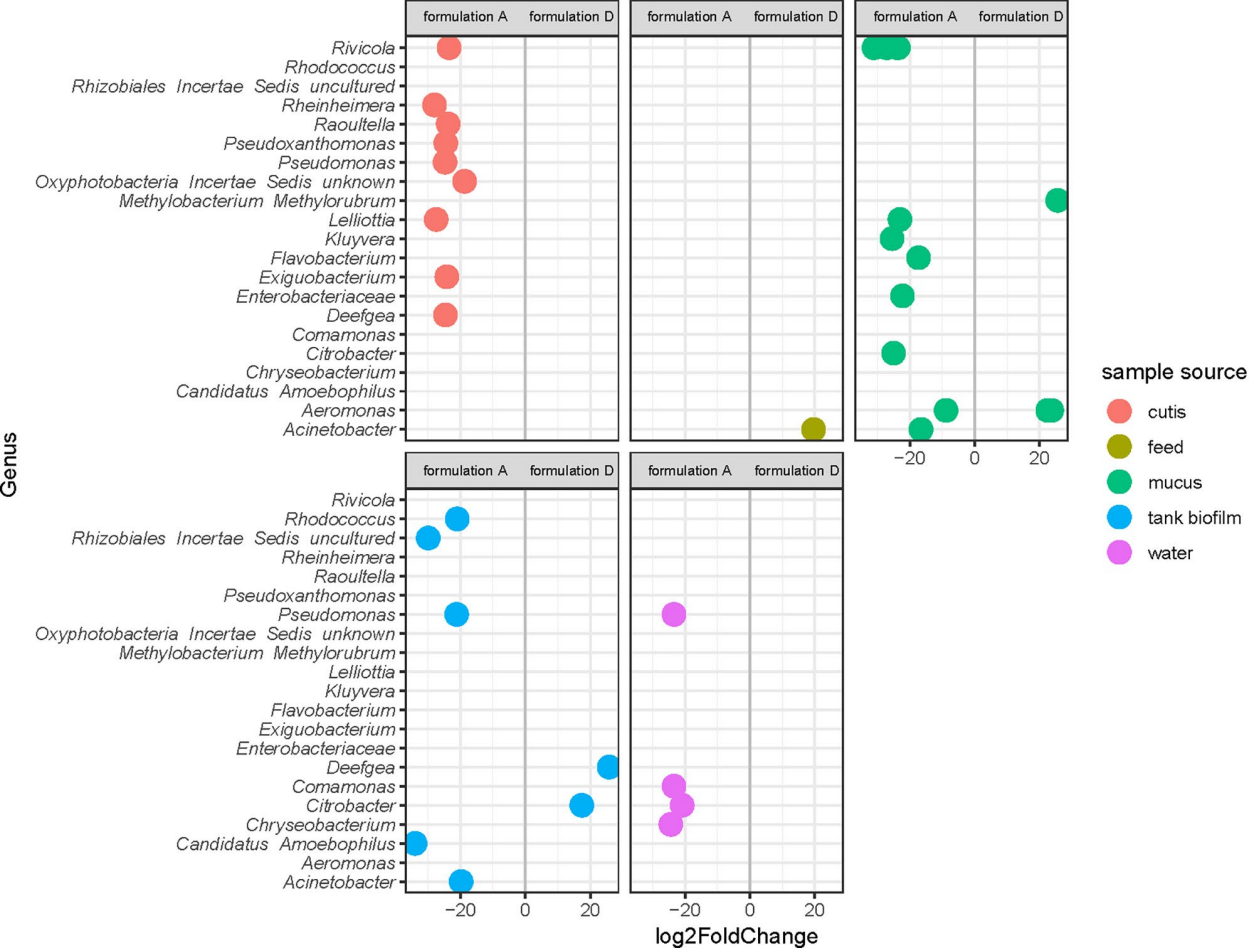
Differential abundance of ASVs (assigned at the genus level) in cutis, feed, mucus, tank biofilm, and water samples considering feed formulation D vs feed formulation A

A sample subset was used, in order to compare the effect of different feeding formulation for each sample source, thus comparing “CUTIS_A”, “CUTIS_D”; “FEED_A”, “FEED_D”; “MUCUS_A”, “MUCUS_D”; “WATER_A”, “WATER_D”;“TANK BIOFILM_A”, “TANK BIOFILM_D”, and accounting for 46 samples and 2948 taxa (Table 1; Fig. 6).

**Table 1:** Differential abundances of genera among different sample sources and feeding formulation groups (A vs D)

Genus	Group A	Group D
*Acinetobacter*	mucus, tank biofilm	feed
*Kluyvera*	mucus	
*Citrobacter*	mucus, water	tank biofilm
*Rivicola*	mucus, cutis	
*Lelliottia*	mucus, cutis	
*Flavobacterium*	mucus	
*Aeromonas*	mucus	mucus
*Methylobacterium*		mucus
*Rheinheimera*	cutis	
*Pseudomonas*	cutis, water, tank biofilm	
*Deefgea*	cutis	tank biofilm
*Pseudoxanthomonas*	cutis	
*Exiguobacterium*	cutis	
*Raoultella*	cutis	
*Chryseobacterium*	water	
*Comamonas*	water	
*Ca.* Amoebophilus	tank biofilm	
*Rhodococcus*	tank biofilm	

Overall, we found that control (feed formulation A) samples had a higher number of genera with a positive differential abundance compared to those related to feed formulation D.

Considering the feed samples, only one ASV assigned to the genus *Acinetobacter* (*Proteobacteria*; *Gammaproteobacteria*; *Pseudomonadales*; *Moraxellaceae*) showed a significant differential abundance compared to feed formulations A and D, being more abundant in D.

*Citrobacter* and *Deefgea* revealed to be significantly more abundant in the biofilm of the tank where feed formulation D was administered, but significantly more abundant in the mucus, water (*Citrobacter*), and cutis samples (*Deefgea*) related to feed formulation A.

**Fig. 7:**
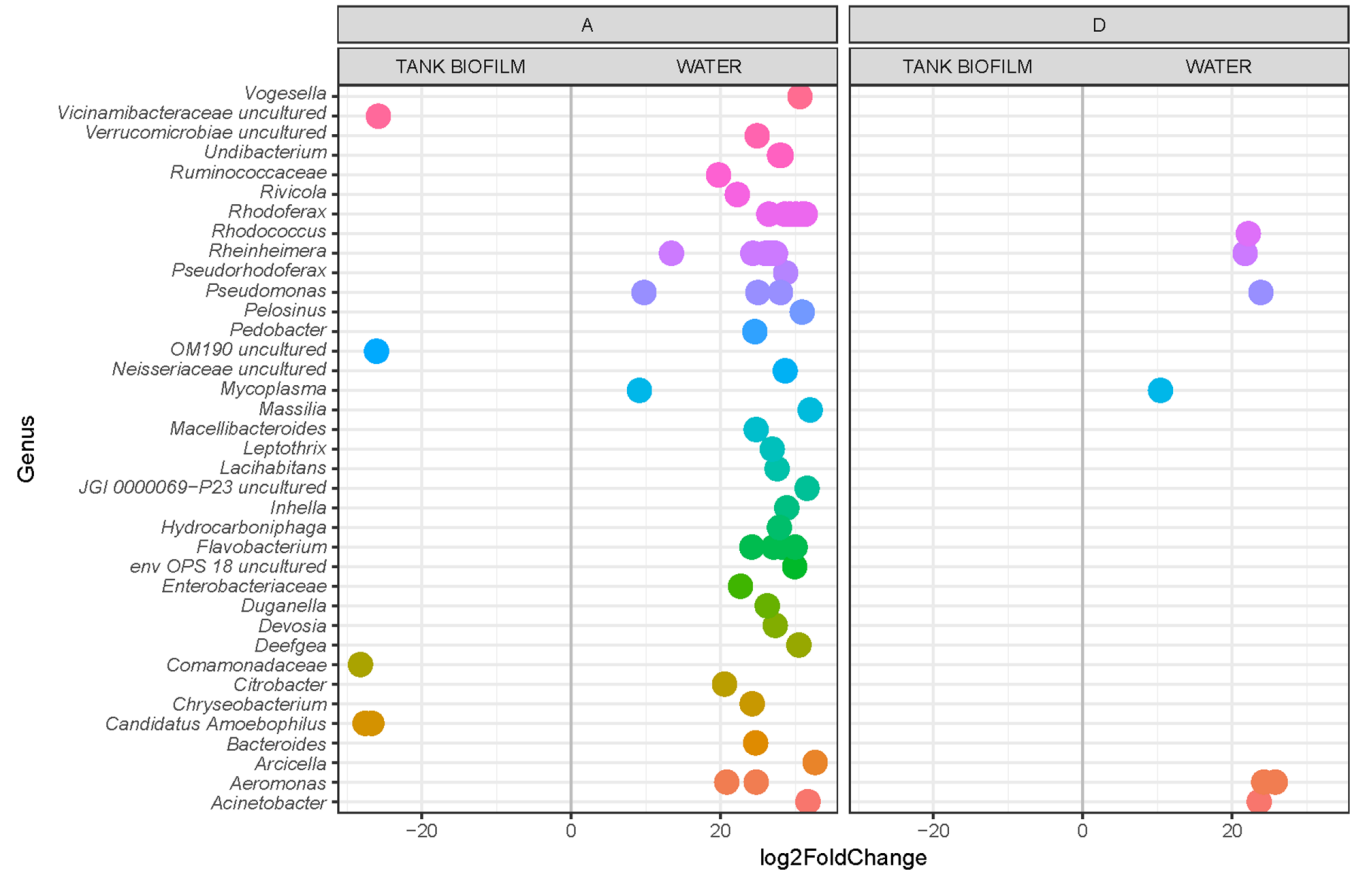
Differential abundance of ASVs (assigned at the genus level) in water and tank biofilm samples considering feed formulations A and D

On the other hand, when we compared the two different water sample sources, namely tank biofilm and water samples, for the two feeding formulations (A and D), we observed a higher number of genera that were significantly more abundant in water than in tank biofilm samples for both feeds administered. Moreover, in tanks where feed formulation A was administered, the number of genera that varied positively was higher than that in D (Fig. 7).

**Fig. 8:**
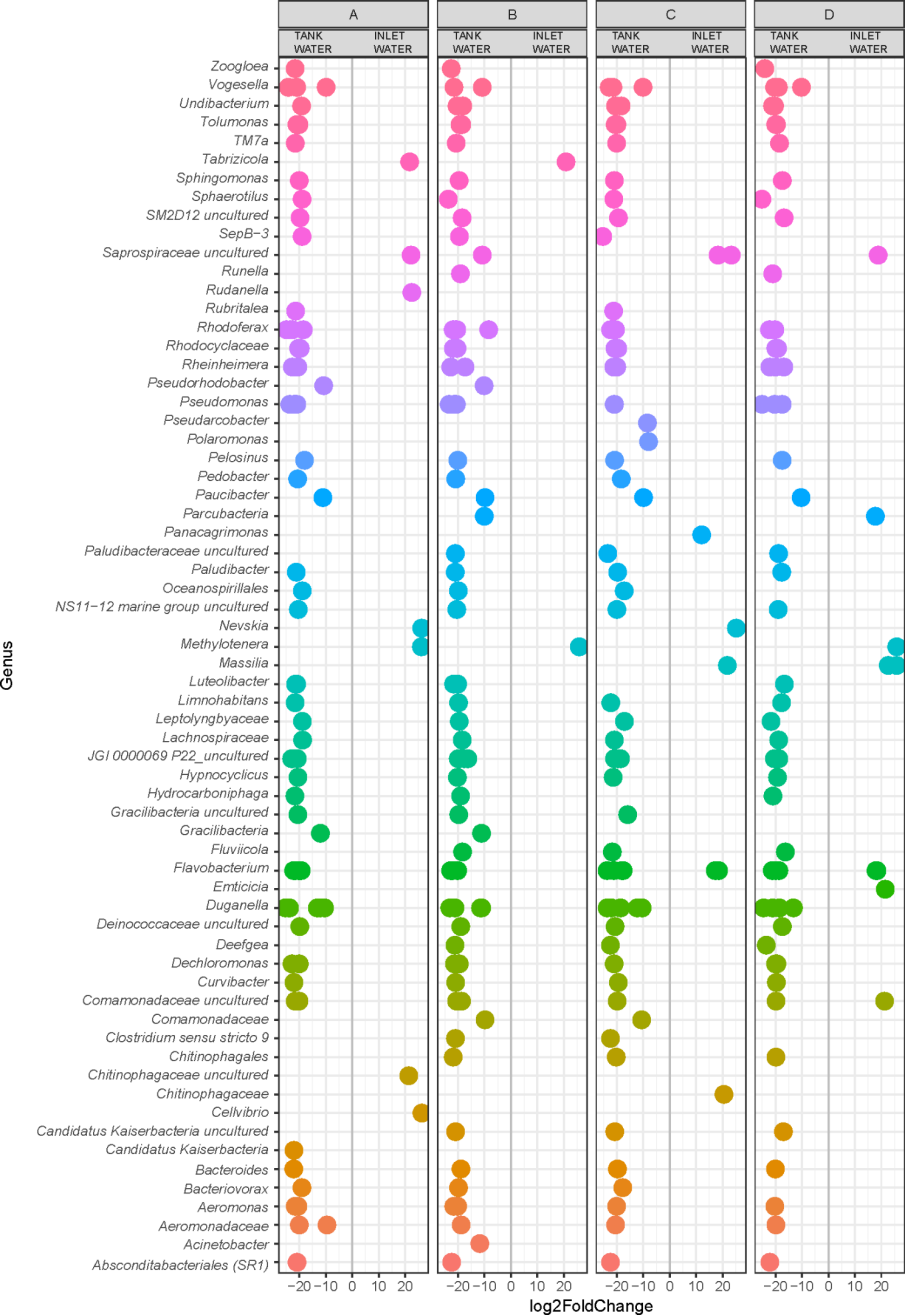
Differential abundance of ASVs (assigned at the genus level) in inlet (before entering the tank) and tank water samples considering feed formulations A, B, C, and D

To evaluate the enrichment of specific taxa, if any, in tank water compared to inlet water (before entering the tank), the differential abundance of ASVs, assigned at the genus level, was also calculated. To do so, we included all the feeding formulations investigated in the case of water samples (A, B, C, and D) (Fig. 8).

Overall, we counted 65 genera that showed a significant difference in relative abundance compared to inlet and tank water, with most genera falling in the tank water group. This was true irrespective of the feeding formulation administered.

## Discussion

A significant challenge for sustainable aquaculture is maintaining a low footprint in the environment and profit farming [2]. Insect-based novel feed formulations are promising solutions and in our case study, we had the opportunity to investigate the effects of full replacement of dietary fishmeal with insect meal from *Tenebrio molitor* on rainbow trout farming.

Taking advantage of the previous data collected in the same trial of rainbow trout gut and skin microbiome [16], we analyzed together the microbial communities characterizing water, tanks, feed, and fish cutis and gut mucus using high-throughput DNA sequencing techniques. Changes in aquaculture-related microbiomes have an impact on farm and animal conditions and performance, which often tips the balance between farm profit and failure.

Here, we discuss the results first focusing on the environment-associated and host-associated microbiomes and then in the light of aquaculture as a holobiome.

### A focus on water ecosystem and the aquaculture built environment

Despite an array of studies evaluating fish gut microbiomes and health, there is a lack of information on how the microbiome of the built environment contributes to fish health and aquaculture system equilibrium. The built environment encompasses all the environments that humans have constructed, including buildings, cars, public transport, other human-built spaces [14], and aquaculture plants [11, 34]. Built environments harbor unique microbial communities, different from those found in other environments on Earth. The aquaculture plant investigated in this study is an experimental-scale facility that uses groundwater to farm fish in a flow-through open system.

Thus, natural (groundwater) and constructed (aquaculture plant) environments are interconnected. In the flow-throw open system, the flow has a direction from the water upstream to the downstream tanks, where fish are farmed, and feed is administered.

In our study, we collected water samples from three different sampling dates, at the beginning, at the intermediate phase, and at the end of the trial. We had the opportunity to sample inlet water (before entering the tanks) and water tanks for the control diet (A) and diet with 100% substitution with insect meal, but also diets with increasing percentages of substitution level (B: 25% and C: 50%). Overall, the microbiological and chemical characteristics recorded in our study showed high-quality water, with low ammonia levels and no diseased fish along the trial.

No significant differences were recorded between the different feeding formulations administered, proving that substitution with an insect-based meal does not affect water quality. An exception is represented by the bacterial load measured by heterotrophic plate count at 22 °C, which was significantly lower in tanks where formulation D was administered (full replacement of fish meal with *T. molitor* larvae meal) compared to formulation A (control). Moreover, as expected, the inlet water showed a significantly lower number of CFUs (both grown at 22 and 37 °C) than tank water, irrespective of the sampling date.

Nevertheless, temporal variation (considering sampling date) was reported when measuring nitrogen compounds and bacterial load by 16S rDNA qPCR assay. The bacterial load on the third sampling date was significantly higher than that on the first and second sampling dates. Marmen et al. [35] demonstrated that the structure of the aquatic bacterial communities in the aquaculture system investigated was explained primarily by natural seasonality, whereas aquaculture-related parameters had only a minor explanatory power. However, in our case study, we cannot say if this may be due to the time passed from the beginning of the trial and/or a seasonality effect, considering that the first sampling date was in spring and the last at the end of summer. The discrepancy between the bacterial load measurements assessed via culture-based methods and DNA-based methods is not unexpected, since the former provides evidence of metabolically active heterotrophic bacteria, whereas the latter detects the DNA of both live and dead bacteria, culturable or not. Thus, these complementary methods suggest that there is a driver of changes that can be seen on the sampling date, but also that differences in microbial taxa may exist for different tanks.

In line with other recent studies [34], the overall composition of the water community from the natural source spring through to the outflow from the farm was stable and not significantly different despite variations in water quality parameters among sampling dates. Overall, water showed astounding microbial diversity compared to host-associated and feed samples, and considering all the alpha diversity metrics tested, water samples, in particular inlet water, had the highest alpha diversity, followed by tank biofilm samples. This is not surprising, as growing evidence has reported the unique microbial biodiversity of water, spanning from natural to artificial aquatic ecosystems and from freshwater to marine resources [36–39].

The beta-diversity analyses highlighted how the microbial communities characterize sample sources, differentiating water-related samples (water and tank biofilm) from host-associated (cutis and gut mucus) and feed samples.

At this point, we wondered whether differences in the abundance of specific taxa existed in the inlet source water and water present in the rearing tanks. Notably, we reported a higher number of genera that significantly increased in relative abundance in tank water than in inlet water. Although they represent preliminary results, given the low number of inlet water samples, they suggest a contribution of farmed fish in shaping the structure of microbial communities in terms of the abundance, and presence/absence of specific taxa.

Many of the genera differentially abundant are typical of freshwater ecosystems, such as *Tabrizicola* (*Rhodobacteraceae*), previously described as a purely chemotrophic, with chlorophyll-dependent phototrophy recently included in the description [40]; *Rudanella* (*Spirosomaceae*) found in air and activated sludge samples [41]; the methylotrophic *Methylotenera* (*Methylophilaceae*), first isolated from Lake Washington sediment [42].

Nevertheless, when we investigated the microbial communities harbored by water in tanks considering different feeding formulations, we observed significant differences in the abundance of specific taxa.

*Chryseobacterium* species (*Weeksellaceae*) were significantly more abundant in the tank water samples where the control diet (A) was administered. These chemoorganotrophic bacteria can be recovered from different environments (soil, freshwater, drinking water, lactic acid beverages, marine sediment and permafrost) and are associated with a multitude of animals (midgut of mosquitoes, cockroach guts, millipede feces, penguin guano, gut homogenates of freshwater copepods, bird feathers, cow’s milk, raw meats, and chicken) [43–46]. *Chryseobacterium* spp. have been recovered from the mucus of apparently healthy fish; however, they are sometimes considered spoilage organisms [47]. In our study, *Chryseobacterium* sequences were also highly abundant in the cutis samples.

The higher abundance of *Citrobacter* in water sample A (also detected in tank biofilm D and mucus A) deserves note, especially in light of the interplay between host-associated and environmental microbial communities. *Citrobacter* is a genus of gram-negative coliform bacteria in the family *Enterobacteriaceae* that can be found in soil, water, and wastewater but is also associated with fish. *C. freundii* strains have inducible ampC genes encoding resistance to ampicillin and first-generation cephalosporins. In addition, isolates of *Citrobacter* may be resistant to many other antibiotics because of their plasmid-encoded resistance genes [48]. Thus, monitoring *Citrobacter* in different compartments of aquaculture plants may be an advisable strategy.

In tank biofilms, *Acinetobacter*, *Pseudomonas*, *Rhodococcus*, and *Candidatus* Amoebophilus were significantly more abundant when feeding formulation A was administered. Interestingly, *Ca*. Amoebophilus (*Cytophagales*; *Amoebophilaceae*) is likely to be a symbiont of the amoebae. Free-living amoebae, such as *Acanthamoeba* spp., are ubiquitous protozoa that can be found in diverse habitats, such as soil, marine water, freshwater, and in many engineered environments. *Ca*. Amoebophilus was recorded only in water and tank biofilm samples. Conversely, the evidence that *Citrobacter* was found to be more abundant in tank biofilm samples belonging to feed formulation D (and in water A, as mentioned previously) suggests that it is not dependent on the feed formulation administered, but on other conditions still undefined. *Deefgea* were also found to be significantly more abundant in tank biofilm D. The presence of *Deefgea* in *O. mykiss* facilities is in line with previous studies that detected this bacterium in the gut and cutis of rainbow trout ([49] and [16] respectively), suggesting water-fish exchange and interconnection.

Supporting our evidence, the study of Minich et al. [50] highlighted the role of the microbiome in the aquaculture environment, analyzing tank water, tank side, inlet water pipe, air stones, and air diffusers along with the feed used. Furthermore, they proposed that the aquaculture built environment may originally be colonized by animal excrement, including mucus, along with environmental sources, such as water, with their peculiar microbial communities. The built environment microbiome can influence the microbial communities of the animal hosts residing there. Understanding the extent to which an animal’s microbiome can be influenced by its surroundings and then associated with a phenotype, such as fish health, will be important for experimental design where microbiome readout is a standard measure.

### Host-associated microbial communities

Both intestinal and skin microbial communities are important for preserving host health. In humans, several studies have demonstrated a bidirectional link between gut dysbiosis and an imbalance in skin homeostasis [51]. Unlike the fish gut microbiome, which has been widely investigated over the last decade in both marine and freshwater species, our knowledge of the skin microbiome remains limited. Fish skin harbors a complex and diverse microbiota that is in constant contact with the external environment (water), which is more susceptible to changes than the gut environment [52].

In the present study, the skin microbial community compositions of dietary groups A and D displayed clearly distinctive features. In accordance with previous studies in fish, the skin microbiome differed markedly from the bacterial communities in the surrounding water [52, 53], except for the *Pseudomonas* genus, which was highly abundant in the skin, water, and tank biofilms of the control group.

At the genus level, trout fed insect meal showed a decrease in the relative abundance of *Rheinheimera*, *Pseudomonas*, *Deefgea*, *Pseudoxanthomonas*, *Exiguobacterium*, and *Raoultella*. All of these, with the exception of *Exiguobacterium*, belong to the phylum *Proteobacteria*. In general, *Proteobacteria* is the predominant phylum in the fish skin microbiome; in particular, *Gammaproteobacteria* class is dominant in the skin of teleosts living in temperate waters, including trout [10, 16, 54]. A decrease in *Proteobacteria* and specifically *Deefgea* genus in the skin-associated microbiome confirmed our previous findings in trout fed *T. molitor* larvae meal [16]. *Deefgea* is a member of the family *Neisseriaceae* and it has been detected in both healthy and unhealthy fish [55]. Unfortunately, little information relating to *Deefgea* is available since only two species belonging to this genus have been isolated so far, i.e., *Deefgea rivuli* and *Deefgea chitinilytica* [56, 57]. Because several other bacterial taxa from the *Neisseriaceae* family have been described to be chitin-hydrolyzing species, it is suspected that *D. chitinilytica* has a similar function as well. This raises the question of whether the presence of chitinolytic bacteria at the skin level can cause diseases such as lesions of the shrimp exoskeleton, which mainly consists of chitin. For this reason, *Deefgea* may have an important role, and has been listed among opportunistic taxa that may be involved in skin infections of aquatic organisms.

In contrast, *Pseudomonas* species are opportunistic bacteria that are naturally resistant to the beta-lactam group of antibiotics and are responsible for septicemic diseases among freshwater fish. Therefore, the decrease in the skin mucus of trout fed with insect meal was a desirable effect. Recent studies have reported that insect meal may influence the immune system of fish at the epidermal mucus level [58, 59]. Interestingly, in a study by Hidalgo et al. [59], an increase in alkaline and acid phosphatase activity was detected in the skin mucus of tenches (*Tinca tinca*) fed different types of insect meal, including *Tenebrio molitor* meal. Since both enzymes are commonly present in the epidermal mucus of fish and have bactericidal activity [60], it was hypothesized that the fish immune system could have been indirectly stimulated by chitin or by other insect components, such as lauric acid [61].

Similarly, at the gut mucosa level, *T. molitor* meal inclusion in the diet led to a significant reduction in gut *Proteobacteria*, predominantly belonging to the class *Gammaproteobacteria*. In line with our previous studies, the abundance of *Citrobacter* and *Kluyvera* genera was significantly reduced in the resident intestinal microbiome of trout fed insect meal, whereas the relative abundance of *Aeromonas* was not affected by diet [16, 62]. The presence of *Citrobacter* genus, as mentioned in the previous paragraph, is worth mentioning, since it includes potential pathogen species, such as *C. freudii* and *C. braakii*, which are Gram-negative bacteria responsible for gastroenteritis and hemorrhagic septicemia in rainbow trout and cyprinids. Furthermore, with respect to the control fish group, feeding insect meal resulted in lower abundance of the *Acinetobacter* genus, another potential pathogen in aquaculture, commonly known as a microorganism that transmits the antibiotic resistance genes. Fish mortality caused by several *Acinetobacter* species, such as *A. baumannii*, *A.lwoffii*, *A. johnsonii*, and *A. calcoaceticus*, has been well documented in rainbow trout [63] and this genus has been classified as harmful for fish intestine. Similarly, *Flavobacteria*, a group of commensal bacteria and serious fish pathogens, were adversely affected by an insect-based diet. Flavobacterial diseases in fish are caused by multiple species within the family *Flavobacteriaceae*, and they are responsible for devastating losses in farmed fish stocks. In the case of acute flavobacteriosis, the mortality rate can be as high as 70%, and survivors may suffer poor growth and spinal abnormalities [64]. For instance, *Flavobacterium psychrophilum* is the etiological agent of coldwater disease and rainbow trout fry syndrome, which cause 50% or greater mortality [65]. Therefore, our findings on the intestinal-associated microbiome indicate that feeding trout with insect meal has a positive effect by inhibiting the growth of potential gram-negative bacterial pathogens.

### Aquaculture as a holobiome

One of the key points of aquaculture ecosystem health is microbial diversity, with HTS technologies increasing our understanding of the role microorganisms play in the health of the ecosystem and hosts. A better understanding of microbial–host interactions will help avoid or manage dysbiosis in aquaculture systems, with the final aim of improving productivity. One of the crucial aims of this study was to investigate shifts in microbial diversity across aquaculture ecosystems.

The inlet water represents the background microbial community entering the tanks. Feed is constantly administered to fish in the tank, carrying out its own microbial contribution. Water is the connection between the surface of the tank (biofilm) and the fish (cutis and gut mucus). All the analyses carried out, spanning from the Venn diagram to alpha diversity and differential abundance analyses, converged in one of the hallmarks of aquatic ecosystems: extreme biodiversity, compared to mucus and feed, which are the least diverse. It is well documented that OTU richness and phylogenetic diversity are higher in healthy water environments [66–68] and increasing evidence supports the fact that the water-associated bacterial microbiome is commonly more diverse (alpha-diversity) than the vertebrate and invertebrate host-associated microbiome [69, 70], showing a clear host-associated selective pressure on the bacterial community.

Considering all the samples collected, only eight features were shared among the different sample sources, and core microbiome analysis revealed the presence of a highly reduced core composed of *Aeromonas* spp. Other studies on the ecology of aquaculture systems have attempted to identify a core microbiome, but they are still not conclusive [71]; however, on a broader scale, freshwater fish have been shown to harbor a high abundance of *Aeromonas* species, whereas the intestinal microbiome of marine fish is dominated by the genus *Vibrio* [72]. *Aeromonas* are known inhabitants of aquatic environments; therefore, fish are common sources for isolating these microorganisms. They are recognized as emerging pathogens because they colonize the host and cause diseases. However, contact with fish and other aquatic animals develops in a continuous and almost inevitable manner and this does not necessarily evolve in pathogenesis [73].

Through network analysis, we showed that the major driver of the microbial community structure is the sample source, with the main differences detected between environmental and host-associated samples. Thus, the different feed formulations did not seem to affect the environment (water and tank biofilm) or fish (cutis and gut mucus). Nevertheless, it was only looking at finer differences (the differential abundance analysis) that it was possible to reveal an enrichment/impoverishment in specific taxa by comparing the samples belonging to the control diet (A) and the samples belonging to the insect-based diet (D).

The increased interest in insects is related not only to their use as raw materials for feed formulation but also as food, as described in more than 150 research papers published on this topic over the last few years (for a review see [74]). This high number of publications represents an indicator of the great potential of insects as feed and food, as well as the need to steer Westerners toward insect-based food acceptance [75]. Furthermore, insect-based feed and food contribute to the microbial community, with marked variations in microbial load and diversity, as well as stable and species-specific microbiomes for some of the most popular edible insect species, such as *T. molitor* [76, 77].

In different contexts, microbial communities have been proposed as valuable signatures [78], peculiar to specific ecosystems and conditions. Thus, such microbial signatures can be exploited to predict the changes occurring in the microbial consortia over time, their role, and their effects on the environment and on the hosts, and therefore on human health.

Our research highlights the interconnection between the environment microbiome and the host-associated microbiome. It proposes the potential of tuning microbiota composition using innovative raw materials like insect meal, as a promising approach for sustainable aquaculture. The results hold significance for researchers and aquaculture experts, as they provide valuable insights for developing healthy and sustainable aquaculture practices, particularly when formulating new feeds.

Consequently, a broader understanding of aquaculture ecosystems as holobiomes is necessary to drive the sustainability of fish and environmental health. In the realm of Global Health concepts towards sustainability in aquaculture, contemporary studies are in favor of environmental biodiversity that encourages more diverse microbiomes [79, 80], eventually resulting in a more resilient system and healthier farmed species. Such studies will pave the way for sufficient knowledge to modulate the microbiome in artificial ecosystems.

## Conclusions

Understanding how the aquaculture microbiome is shaped is rather challenging, owing to the complexity of microbial community assembly, but it is pivotal for efficient and sustainable farming. Sustainable aquaculture requires the replacement of FM with novel feed formulations. However, most research efforts have focused on the effects on fish growth and the fish gut microbiome. Water plays a crucial role in connecting the surfaces of this peculiar built environment, that is, the tank (biofilm) and fish (cutis and intestinal mucus). Omic exploration of the water-fish interface exposes patterns otherwise undetected. The research presented here offers a step toward modulating the aquaculture environment and its microbiome for beneficial outcomes.

### Electronic supplementary material

Below is the link to the electronic supplementary material.

Supplementary Material 1

Supplementary Material 2

## Data Availability

The datasets generated and analyzed for this study can be found in the European Nucleotide Archive (ENA) under the accession number PRJEB56592 and PRJEB38845.
