# Supplementary material for: Aquaculture ecosystem microbiome at the water-fish interface: the case-study of rainbow trout fed with Tenebrio molitor novel diets
Source: BMC Microbiol. 2023 Sep 6;23:248. doi: 10.1186/s12866-023-02990-y (PMC10481543; doi:10.1186/s12866-023-02990-y)

***Supplementary Materials***

**Aquaculture ecosystem microbiome at the water-fish interface: the case-study of rainbow trout fed with *Tenebrio molitor* novel feed diets**

**Antonia Bruno^1*^, Anna Sandionigi^2^, Antonella Panio^3^, Simona Rimoldi^4^, Flavio Orizio^1^, Giulia Agostinetto^1^, Imam Hasan^4^, Laura Gasco^5^, Genciana Terova^4^ ^†^, Massimo Labra^1†^**

**Supplementary Table S01.** Ingredients (% as fed) of the experimental diets.

| **Ingredient** | **A** | **B** | **C** | **D** |
| --- | --- | --- | --- | --- |
| Fishmeal 65 (Peruvian) | 20 | 15 | 10 | 0 |
| Tenebrio molitor larvae meal | - | 5 | 10 | 20 |
| Soy protein concentrate | 18 | 18 | 18 | 18 |
| Wheat gluten | 7.75 | 7.4 | 7.4 | 7.06 |
| Corn gluten | 8 | 8 | 8 | 8 |
| Soybean meal 48% | 7 | 7 | 7 | 7 |
| Wheat meal | 14.23 | 14 | 14.23 | 13.8 |
| Sardine oil | 4.3 | 4.26 | 4.2 | 4.1 |
| Soybean oil | 8.6 | 8.52 | 8.4 | 8.2 |
| Rapeseed oil | 8.6 | 8.52 | 8.4 | 8.2 |
| Soy lecithin | 0.5 | 0.5 | 0.5 | 0.5 |
| Vit-Min Premix | 1 | 1 | 1 | 1 |
| Antioxidant | 0.2 | 0.2 | 0.2 | 0.2 |
| Sodium propionate | 0.1 | 0.1 | 0.1 | 0.1 |
| Monocalcium phosphate | 0.52 | 0.92 | 0.92 | 1.72 |
| L-Arginine | - | - | - | 0.1 |
| L-Lysine | - | 0.3 | 0.3 | 0.6 |
| L-Tryptophan | 0.05 | 0.08 | 0.1 | 0.12 |
| DL-methionine | 0.15 | 0.2 | 0.25 | 0.3 |
| Celite® | 1 | 1 | 1 | 1 |

**Supplementary Data S01.**

- **Bacterial load of water samples: 16S rDNA quantification**

Bacterial DNA copies are reported in Fig. S01 and Table S02. Melting temperatures (Tm) measured after the dissociation stage varied from 83.8–85.9°C.

Statistical analysis revealed the low impact of filter type used (P = 0.731) on bacterial load and, for this reason, the subsequent analyses were carried out considering only the samples filtered with 0.2 µm filters. qPCR assay showed that a higher quantity of DNA was detected in water samples belonging to the third sampling date. Statistical analyses confirmed the hypothesis that the difference between the third and second and between the third and the first sampling date was significant (P < 0.001). Moreover, no significant difference was measured comparing inlet water bacterial load of the three sampling dates. Inlet water showed lower 16S DNA counts than tank water. Analysing the differences considering the quantity of bacterial DNA of water belonging to tanks where different feeding formulations were administered, no effect was detected (P = 0.316). As regards water biofilm samples, obtained during the third sampling campaign, the same result was observed (P = 0.204).

- **Bacterial load of water samples: CFUs enumeration**

CFU/mL of water samples are reported in Fig. S02 and Table S03. Considering CFUs enumeration according to D. Lgs. n. 31 of 2 February 2001 at both 22 and 37 °C, inlet water samples showed a significantly lower number of CFUs (P <0.001), despite the sampling date.

Considering CFUs enumeration after seven days of incubation, we confirmed the significant difference between inlet water bacterial load and water tanks bacterial load, for both 22 °C and 37 °C growth temperatures (P <0.001). We observed that a significant difference existed between control tanks (A, no insect feeding) and the tanks of maximum substitution (D, 100% insect meal), for growth temperature = 22 °C (P < 0.001).

Statistical analyses also revealed that there was no significant difference in CFUs enumeration data considering sampling date (P = 0.22).

Representative colonies (each morphotype represented by at least 1 CFU for each petri) were selected for isolation and purification. 50 isolates were characterised by 16S rRNA gene sequencing. Briefly, crude cell lysate was obtained from each bacterial isolate. Amplicons of the genes 16S rRNA were obtained with the primers 27F/1492R and Wonder Taq Thermostable DNA polymerase© (EuroClone S.p.A.) kit. The reaction mix was composed as follows: 0.25 µl of Wonder Taq, 4 µl of Wonder Taq Reaction Buffer, 0.2 µl each primer [100 µM], 2 µl of DNA sample and Milli-Q water to reach the volume of 20 µl.

Amplicons were purified from agarose using EuroGOLD Gel Extraction Kit © (EuroClone S.p.A.) following manufacturer’s protocol.

Products were submitted for sequencing to Eurofins Genomics (https://www.eurofinsgenomics.eu/).

The DNA strands were bidirectionally sequenced. All obtained sequences were edited with UniPro UGENE (Okonechnikov et al., 2012), aligned, and used as a query to verify samples identity in Silva SSU 138 Database (<https://www.arb-silva.de/>).

Environmental bacteria identification through full-length 16S rDNA Sanger sequencing revealed the presence of ten different genera (*Acinetobacter, Aeromonas, Bacillus, Chryseobacterium, Duganella, Enterococcus, Flavobacterium, Pseudomonas, Rhodococcus, Vogesella*), nine families (*Enterococcaceae, Moraxellaceae, Flavobacteriaceae, Aeromonadaceae, Bacillaceae, Pseudomonadaceae, Nocardiaceae, Neisseriaceae, Oxalobacteraceae*), eight orders (*Lactobacillales, Pseudomonadales, Flavobacteriales, Aeromonadales, Bacillales, Actinomycetales, Neisseriales, Burkholderiales*), five classes (*Bacilli, Gammaproteobacteria, Flavobacteria, Actinobacteria, Betaproteobacteria*), and three phyla (*Proteobacteria, Firmicutes, Actinobacteria*).

Bacteria assigned to the genus *Aeromonas* were the most abundant and spread, considering the whole sampling campaign.

**Fig. S01. 16S rDNA quantification of tank biofilm and water samples.** Values are expressed as log_2_(DNA counts) considering sample type (different feeding formulations and inlet water) and sampling date.


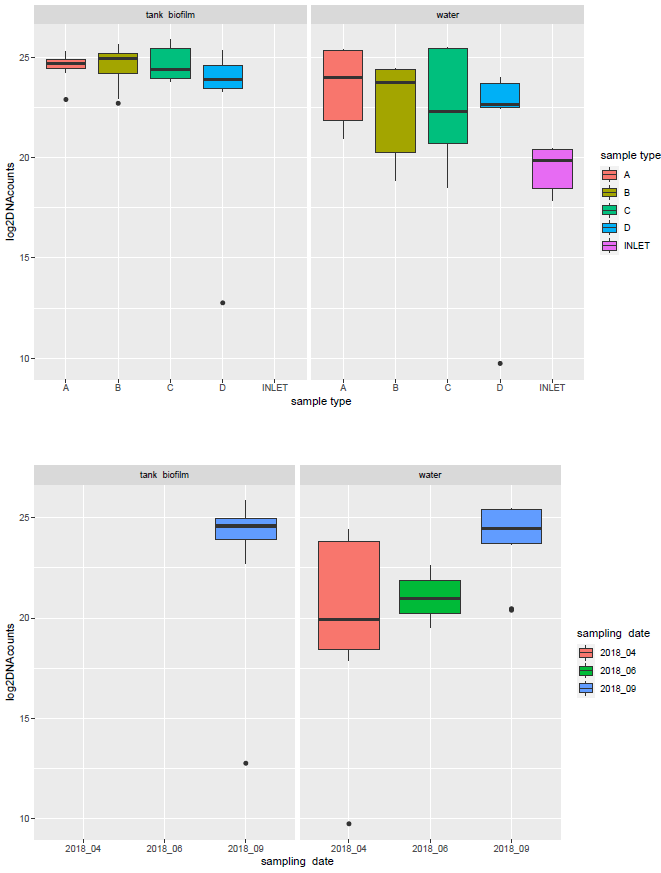


**Supplementary Table S02. 16S rDNA quantification of tank biofilm and water samples.** Values are expressed as log_2_(DNA counts) considering sample type (different feeding formulations and inlet water) and sampling date.

| **Sample type** | **Sample source** | **Sampling date** | **log2DNAcounts** |
| --- | --- | --- | --- |
| A | water | 2018_04 | 24.41 |
| A | water | 2018_04 | 24 |
| A | water | 2018_04 | 20.95 |
| B | water | 2018_04 | 23.96 |
| B | water | 2018_04 | 18.87 |
| B | water | 2018_04 | 23.74 |
| C | water | 2018_04 | 23.63 |
| C | water | 2018_04 | 18.52 |
| D | water | 2018_04 | 9.74 |
| INLET | water | 2018_04 | 17.86 |
| INLET | water | 2018_04 | 18.44 |
| INLET | water | 2018_04 | 18.45 |
| A | water | 2018_06 | 21.9 |
| A | water | 2018_06 | 21.85 |
| A | water | 2018_06 | 21.21 |
| B | water | 2018_06 | 20.28 |
| B | water | 2018_06 | 21.35 |
| B | water | 2018_06 | 20.14 |
| C | water | 2018_06 | 20.96 |
| C | water | 2018_06 | 20.62 |
| C | water | 2018_06 | 20.73 |
| D | water | 2018_06 | 22.56 |
| D | water | 2018_06 | 22.64 |
| D | water | 2018_06 | 22.43 |
| INLET | water | 2018_06 | 19.51 |
| INLET | water | 2018_06 | 19.98 |
| INLET | water | 2018_06 | 19.85 |
| A | water | 2018_09 | 25.4 |
| A | water | 2018_09 | 25.38 |
| A | water | 2018_09 | 25.34 |
| B | water | 2018_09 | 24.46 |
| B | water | 2018_09 | 24.46 |
| B | water | 2018_09 | 24.41 |
| C | water | 2018_09 | 25.45 |
| C | water | 2018_09 | 25.47 |
| C | water | 2018_09 | 25.47 |
| D | water | 2018_09 | 23.98 |
| D | water | 2018_09 | 23.67 |
| D | water | 2018_09 | 23.74 |
| INLET | water | 2018_09 | 20.44 |
| INLET | water | 2018_09 | 20.39 |
| INLET | water | 2018_09 | 20.46 |
| A | tank_biofilm | 2018_09 | 24.96 |
| A | tank_biofilm | 2018_09 | 24.57 |
| A | tank_biofilm | 2018_09 | 24.62 |
| A | tank_biofilm | 2018_09 | 22.9 |
| A | tank_biofilm | 2018_09 | 24.23 |
| A | tank_biofilm | 2018_09 | 24.69 |
| A | tank_biofilm | 2018_09 | 24.32 |
| A | tank_biofilm | 2018_09 | 24.83 |
| A | tank_biofilm | 2018_09 | 24.81 |
| B | tank_biofilm | 2018_09 | 24.94 |
| B | tank_biofilm | 2018_09 | 25.62 |
| B | tank_biofilm | 2018_09 | 25.32 |
| C | tank_biofilm | 2018_09 | 25.44 |
| C | tank_biofilm | 2018_09 | 25.87 |
| C | tank_biofilm | 2018_09 | 25.5 |
| D | tank_biofilm | 2018_09 | 23.3 |
| D | tank_biofilm | 2018_09 | 25.35 |
| D | tank_biofilm | 2018_09 | 24.97 |
| A | tank_biofilm | 2018_09 | 25.25 |
| A | tank_biofilm | 2018_09 | 25.29 |
| A | tank_biofilm | 2018_09 | 24.56 |
| B | tank_biofilm | 2018_09 | 24.64 |
| B | tank_biofilm | 2018_09 | 25.17 |
| B | tank_biofilm | 2018_09 | 24.96 |
| C | tank_biofilm | 2018_09 | 24.35 |
| C | tank_biofilm | 2018_09 | 24.55 |
| C | tank_biofilm | 2018_09 | 24.39 |
| D | tank_biofilm | 2018_09 | 12.76 |
| D | tank_biofilm | 2018_09 | 23.94 |
| D | tank_biofilm | 2018_09 | 23.9 |
| A | tank_biofilm | 2018_09 | 24.97 |
| A | tank_biofilm | 2018_09 | 24.32 |
| A | tank_biofilm | 2018_09 | 24.77 |
| B | tank_biofilm | 2018_09 | 24.21 |
| B | tank_biofilm | 2018_09 | 22.93 |
| B | tank_biofilm | 2018_09 | 22.71 |
| C | tank_biofilm | 2018_09 | 23.79 |
| C | tank_biofilm | 2018_09 | 23.88 |
| C | tank_biofilm | 2018_09 | 23.93 |
| D | tank_biofilm | 2018_09 | 24.6 |
| D | tank_biofilm | 2018_09 | 23.47 |
| D | tank_biofilm | 2018_09 | 23.55 |

**Fig. S02. CFUs enumeration of water samples.** After 7 days of incubation at 22 and 37 °C, considering sample type (different feeding formulations and inlet water) and sampling date. Values are expressed as CFU/mL.


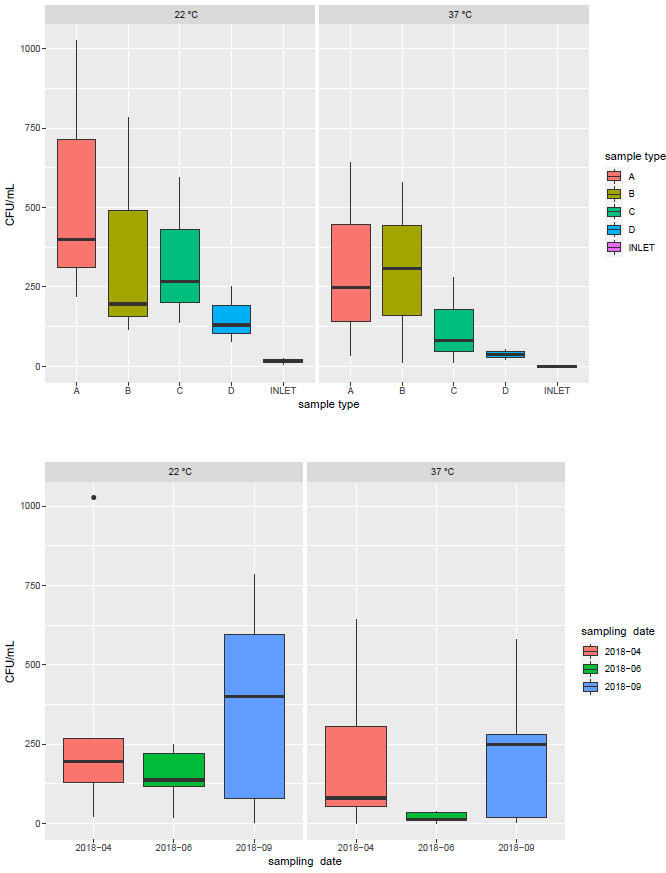


**Supplementary Table S03. CFUs enumeration of water samples.** After 7 days of incubation at 22 and 37 °C, considering sample type (different feeding formulations and inlet water) and sampling date. Values are expressed as CFU/mL and as the average of triplicate measurements.

| **Sample type** | **Sampling date** | **CFU/mL** | **Growth temperature** |
| --- | --- | --- | --- |
| INLET | 2018-04 | 23 | 22 |
| A | 2018-04 | 1027 | 22 |
| B | 2018-04 | 196 | 22 |
| C | 2018-04 | 267 | 22 |
| D | 2018-04 | 130 | 22 |
| INLET | 2018-06 | 18 | 22 |
| A | 2018-06 | 220 | 22 |
| B | 2018-06 | 117 | 22 |
| C | 2018-06 | 137 | 22 |
| D | 2018-06 | 250 | 22 |
| INLET | 2018-09 | 4 | 22 |
| A | 2018-09 | 400 | 22 |
| B | 2018-09 | 784 | 22 |
| C | 2018-09 | 596 | 22 |
| D | 2018-09 | 77 | 22 |
| INLET | 2018-04 | 0 | 37 |
| A | 2018-04 | 643 | 37 |
| B | 2018-04 | 307 | 37 |
| C | 2018-04 | 80 | 37 |
| D | 2018-04 | 54 | 37 |
| INLET | 2018-06 | 0 | 37 |
| A | 2018-06 | 34 | 37 |
| B | 2018-06 | 11 | 37 |
| C | 2018-06 | 13 | 37 |
| D | 2018-06 | 37 | 37 |
| INLET | 2018-09 | 2 | 37 |
| A | 2018-09 | 248 | 37 |
| B | 2018-09 | 580 | 37 |
| C | 2018-09 | 280 | 37 |
| D | 2018-09 | 20 | 37 |

**Supplementary Data S02. Chemical analyses of water samples**

The concentration ​​of phosphorus, nitrites and ammoniacal nitrogen was found near zero or below the detection limit of the analysis performed. Parameters that gave concentration > 0 mg/L or above the sensitivity limit of the test were only nitrates and, as a consequence, total nitrogen.

No significant difference was found, considering different feeds, but a significant difference considering sampling date was reported for nitrates and total nitrogen concentrations for September 2018 and April 2018, respectively (Fig. S03, Table S04).

**Fig. S03. Nitrate and total nitrogen concentrations in water samples.** Samples are plotted considering sample type (different feeding formulations and inlet water) and the three sampling dates.

**
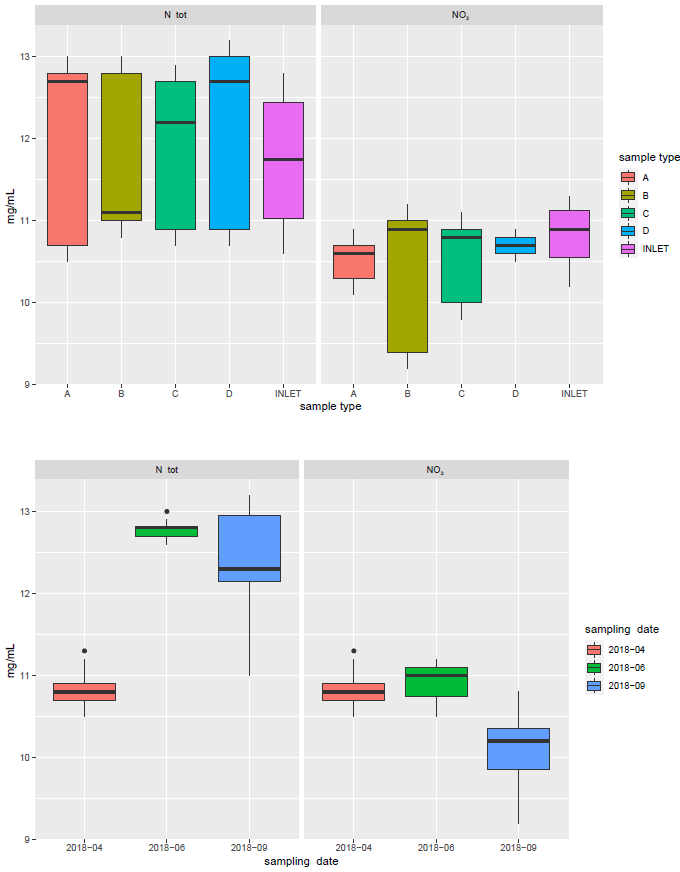
**

**Table S04. Nitrate concentration in water samples.** Measurements were made in each tank considering sample type (different feeding formulations and inlet water) and the three sampling dates. Values are expressed as the average of triplicate measurements.

| **Sample type** | **Sampling date** | **NO3 (mg/L)** | **N total (mg/L)** |
| --- | --- | --- | --- |
| A | 2018-04 | 10.6 | 10.6 |
| B | 2018-04 | 10.9 | 10.9 |
| C | 2018-04 | 10.8 | 10.8 |
| D | 2018-04 | 10.8 | 10.8 |
| INLET | 2018-04 | 10.7 | 10.7 |
| INLET | 2018-04 | 11.2 | 11.2 |
| A | 2018-06 | 10.8 | 12.7 |
| B | 2018-06 | 11.1 | 12.9 |
| C | 2018-06 | 11.0 | 12.8 |
| D | 2018-06 | 10.6 | 12.7 |
| INLET | 2018-06 | 11.1 | 12.7 |
| A | 2018-09 | 10.2 | 12.9 |
| B | 2018-09 | 9.3 | 11.1 |
| C | 2018-09 | 9.9 | 12.2 |
| D | 2018-09 | 10.7 | 13.1 |
| INLET | 2018-09 | 10.3 | 12.3 |

**Fig. S04. Reads distribution plot of water samples.**


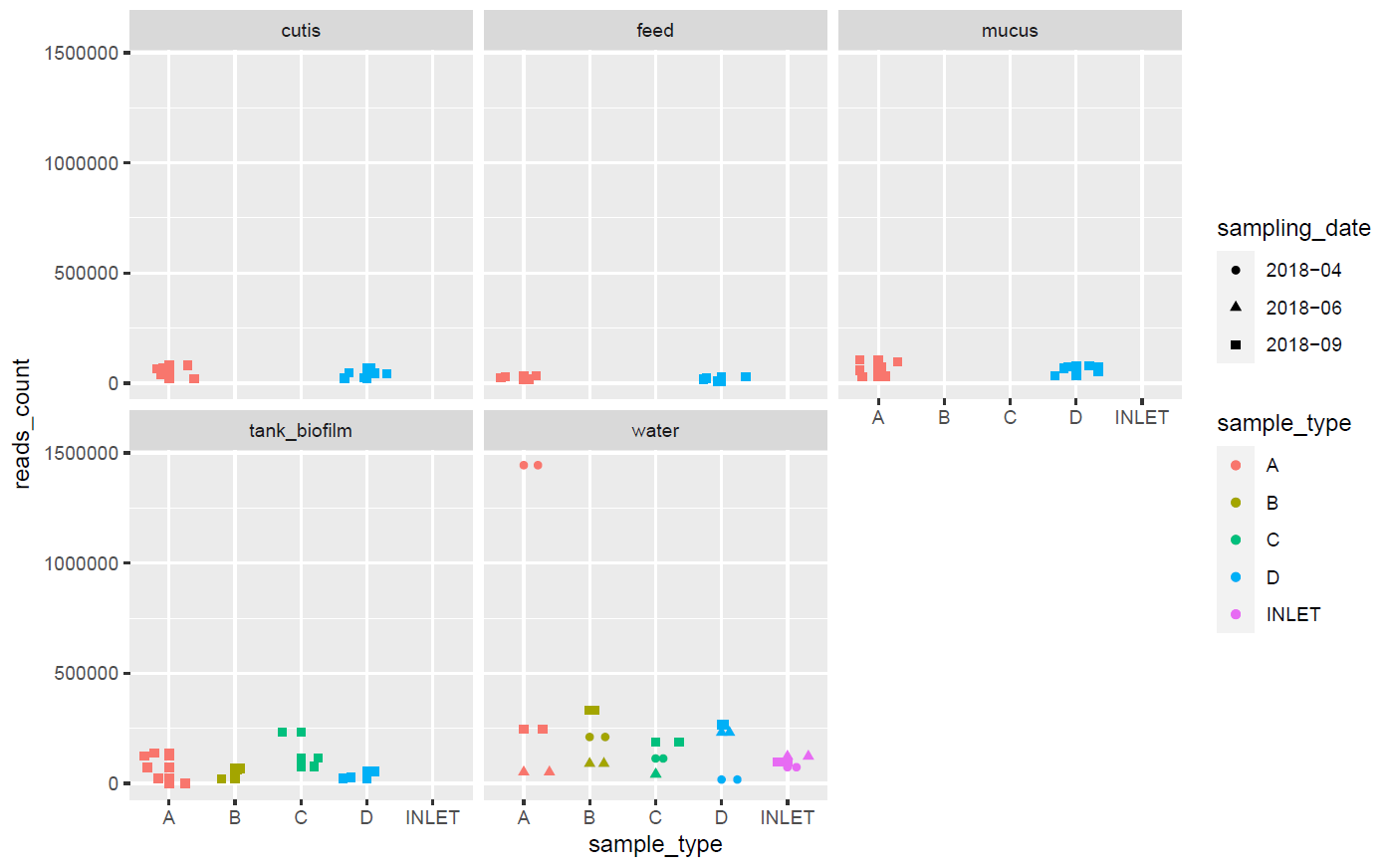


**Fig. S05. Heatmap.** Samples are sorted by sample source. ASVs are assigned to the taxonomic rank of genus.


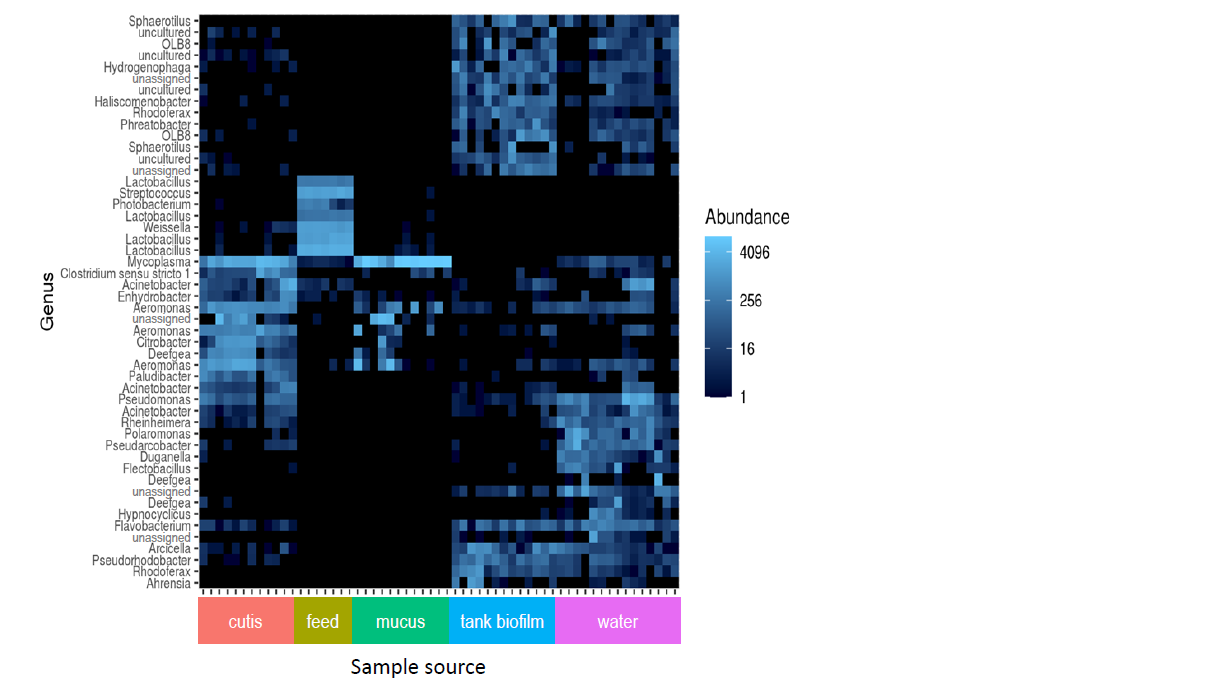


**Table S05. Paired ADONIS test considering sample source.**

| **Sample source** | **Sums Of Sqs** | **Mean Sqs** | **F.Model** | **R2** | **P value** | **P value corrected** |
| --- | --- | --- | --- | --- | --- | --- |
| cutis <-> feed | 3.6291943 | 3.6291943 | 30.309846 | 0.6406668 | 0.000999001 | **0.001498501** |
| cutis <-> mucus | 1.5394493 | 1.5394493 | 9.489275 | 0.3013494 | 0.000999001 | **0.001498501** |
| cutis <-> swab | 3.2896768 | 3.2896768 | 13.496605 | 0.3698044 | 0.000999001 | **0.001498501** |
| cutis <-> water | 2.6264161 | 2.6264161 | 11.291813 | 0.3391769 | 0.000999001 | **0.001498501** |
| cutis <-> water_I | 1.3265383 | 1.3265383 | 6.448093 | 0.3315540 | 0.002997003 | **0.003458080** |
| feed <-> mucus | 3.7996036 | 3.7996036 | 39.597375 | 0.6996327 | 0.000999001 | **0.001498501** |
| feed <-> swab | 3.2474476 | 3.2474476 | 15.935240 | 0.4695779 | 0.000999001 | **0.001498501** |
| feed <-> water | 3.2261397 | 3.2261397 | 17.250644 | 0.5036590 | 0.000999001 | **0.001498501** |
| feed <-> water_I | 1.6001123 | 1.6001123 | 17.378003 | 0.6847664 | 0.013986014 | **0.013986014** |
| mucus <-> swab | 3.6665867 | 3.6665867 | 16.212060 | 0.4134458 | 0.000999001 | **0.001498501** |
| mucus <-> water | 3.5491526 | 3.5491526 | 16.567881 | 0.4295772 | 0.000999001 | **0.001498501** |
| mucus <-> water_I | 1.5273821 | 1.5273821 | 8.746488 | 0.4022023 | 0.001998002 | **0.002724548** |
| swab <-> water | 1.7620456 | 1.7620456 | 6.004195 | 0.2070113 | 0.000999001 | **0.001498501** |
| swab <-> water_I | 0.9099995 | 0.9099995 | 2.957878 | 0.1744250 | 0.002997003 | **0.003458080** |
| water <-> water_I | 0.7029836 | 0.7029836 | 2.393494 | 0.1554874 | 0.007992008 | **0.008562866** |

**Table S06. Paired ADONIS test considering sample type.**

| **Sample type** | **Sums Of Sqs** | **Mean Sqs** | **F Model** | **R2** | **P value** | **P value corrected** |
| --- | --- | --- | --- | --- | --- | --- |
| CUTIS_A <-> CUTIS_D | 0.39847958 | 0.39847958 | 2.5090173 | 0.20057669 | 0.020979021 | 0.04354136 |
| FEED_A <-> FEED_D | 0.03283542 | 0.03283542 | 10.2512549 | 0.67215812 | 0.022977023 | 0.04467754 |
| MUCUS_A <-> MUCUS_D | 0.51788215 | 0.51788215 | 4.8649440 | 0.32727631 | 0.010989011 | 0.03575969 |
| WATER_A <-> WATER_D | 0.24554027 | 0.24554027 | 0.7198788 | 0.15252061 | 0.800000000 | 0.81553398 |
| TBIOFILM_A <-> TBIOFILM_D | 0.37332822 | 0.37332822 | 1.2709165 | 0.20266838 | 0.124875125 | 0.13948817 |
| WATER_A <-> WATER_B | 0.19768093 | 0.19768093 | 0.6195625 | 0.13411714 | 0.700000000 | 0.74242424 |
| WATER_A <-> WATER_C | 0.17463569 | 0.17463569 | 0.5048659 | 0.11207124 | 0.700000000 | 0.74242424 |
| TBIOFILM_A <-> TBIOFILM_B | 0.28806058 | 0.28806058 | 0.8842616 | 0.15027572 | 0.773226773 | 0.81188811 |
| TBIOFILM_A <-> TBIOFILM_C | 0.29340155 | 0.29340155 | 0.9245493 | 0.15605395 | 0.793206793 | 0.81553398 |

**Fig. S06. NMDS plot considering phyla.**


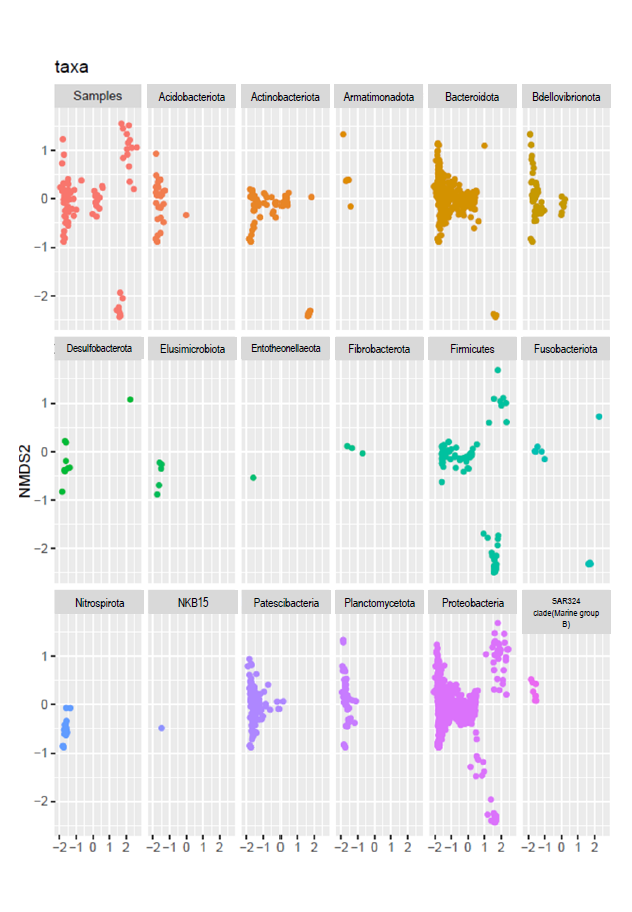


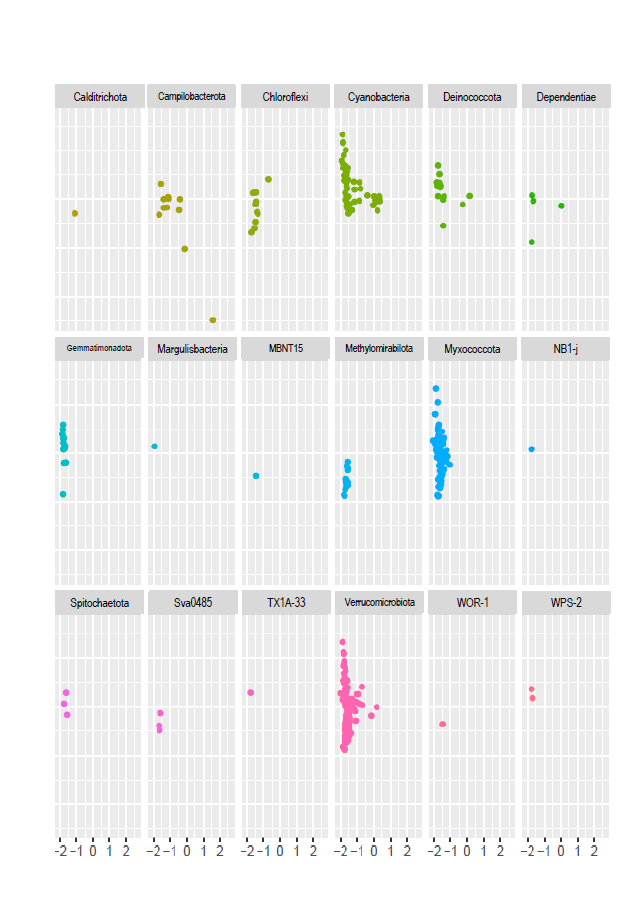

Supplement: Supplementary file 1 — Supplementary Material 1 [file 12866_2023_2990_MOESM1_ESM.docx]
